# Cyclic Boronates Inhibit All Classes of β-Lactamases

**DOI:** 10.1128/AAC.02260-16

**Published:** 2017-03-24

**Authors:** Samuel T. Cahill, Ricky Cain, David Y. Wang, Christopher T. Lohans, David W. Wareham, Henry P. Oswin, Jabril Mohammed, James Spencer, Colin W. G. Fishwick, Michael A. McDonough, Christopher J. Schofield, Jürgen Brem

**Affiliations:** a Chemistry Research Laboratory, University of Oxford, Oxford, United Kingdom; b Queen Mary University of London, London, United Kingdom; c School of Cellular and Molecular Medicine, University of Bristol, Bristol, United Kingdom; d School of Chemistry, University of Leeds, Leeds, United Kingdom

**Keywords:** antibiotic resistance, beta-lactamases, beta-lactams, boronate, carbapenemase, inhibitors, metalloenzymes

## Abstract

β-Lactamase-mediated resistance is a growing threat to the continued use of β-lactam antibiotics. The use of the β-lactam-based serine-β-lactamase (SBL) inhibitors clavulanic acid, sulbactam, and tazobactam and, more recently, the non-β-lactam inhibitor avibactam has extended the utility of β-lactams against bacterial infections demonstrating resistance via these enzymes. These molecules are, however, ineffective against the metallo-β-lactamases (MBLs), which catalyze their hydrolysis. To date, there are no clinically available metallo-β-lactamase inhibitors. Coproduction of MBLs and SBLs in resistant infections is thus of major clinical concern. The development of “dual-action” inhibitors, targeting both SBLs and MBLs, is of interest, but this is considered difficult to achieve due to the structural and mechanistic differences between the two enzyme classes. We recently reported evidence that cyclic boronates can inhibit both serine- and metallo-β-lactamases. Here we report that cyclic boronates are able to inhibit all four classes of β-lactamase, including the class A extended spectrum β-lactamase CTX-M-15, the class C enzyme AmpC from Pseudomonas aeruginosa, and class D OXA enzymes with carbapenem-hydrolyzing capabilities. We demonstrate that cyclic boronates can potentiate the use of β-lactams against Gram-negative clinical isolates expressing a variety of β-lactamases. Comparison of a crystal structure of a CTX-M-15:cyclic boronate complex with structures of cyclic boronates complexed with other β-lactamases reveals remarkable conservation of the small-molecule binding mode, supporting our proposal that these molecules work by mimicking the common tetrahedral anionic intermediate present in both serine- and metallo-β-lactamase catalysis.

## INTRODUCTION

The β-lactam antibiotics remain the most important drug class for the treatment of bacterial infections (1). However, their continued use is jeopardized by the increasing spread of resistance mechanisms, including that mediated by β-lactamases, which, cumulatively, can hydrolyze all classes of β-lactam antibiotics (2). β-Lactamases can be divided into four classes (Ambler classes A, B, C, and D [3]) and manifest considerable sequence and structural diversity as well as different, but overlapping, substrate profiles (4). The serine-β-lactamases (SBLs), classes A, C, and D, likely evolved from the penicillin-binding protein (PBP) targets of β-lactam antibiotics (5–8). Among the SBLs, the extended-spectrum SBLs (ESBLs) are of particular clinical concern. Their ability to hydrolyze extended spectrum cephalosporins and the monobactam aztreonam (AZT) (9) is an important reason for failure of cephalosporin-based therapies (10). Of particular note are the CTX-M enzymes, which have become the most prevalent ESBLs worldwide (11). The SBL carbapenemases, such as variants of class D enzymes OXA-23 and OXA-48, are a growing concern, since they are able to hydrolyze carbapenems, which have often been used as the last line of antibacterial defense (12). Inhibitors of the SBLs include the β-lactams clavulanic acid (CLAV), sulbactam (SUL), and tazobactam (TAZ), which are active against class A β-lactamases (2, 13, 14), and the recently introduced non-β-lactam β-lactamase inhibitor avibactam, which has a broader spectrum of SBL inhibition activity (15, 16). These inhibitors have increased the efficacy of β-lactam antibiotics against SBL-mediated resistance in bacteria, but they are inactive against the Zn(II)-dependent class B metallo-β-lactamases (MBLs), which constitute a structural and mechanistically distinct family of enzymes and exhibit considerable heterogeneity, even among themselves (17). The MBLs are able to hydrolyze all classes of β-lactam except for monobactams (18). The ability of the MBLs to hydrolyze SBL inhibitors, including avibactam (19), is a growing problem in the treatment of infections where both SBL- and MBL-mediated cephalosporin and carbapenem resistance have been acquired (20). To date there are no clinically approved MBL inhibitors.

**FIG 1:**
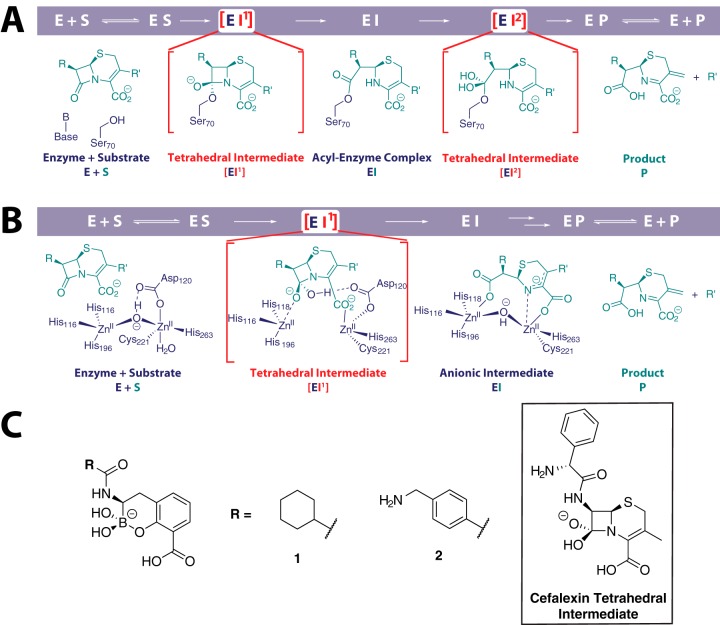
(A and B) Outline mechanism of cephalosporin hydrolysis by serine-β-lactamases (A) and metallo-β-lactamases (B). The small-molecule elements of the first tetrahedral intermediate EI^1^ is common to both mechanisms. (C) Chemical structures of the two cyclic boronates used in this study. The structure of the proposed common tetrahedral intermediate in the serine and metallo-β-lactamase-catalyzed hydrolysis of β-lactam antibiotics is shown for a cephalosporin substrate (cefalexin). We propose that the cyclic boronates mimic this intermediate.

As a consequence of increasing variation in the β-lactamase-mediated resistance to β-lactam antibiotics, the development of broad-spectrum inhibitors of both the SBLs and MBLs is of considerable interest. This is presently perceived to be challenging due to the mechanistic and structural differences between the SBLs and MBLs (Fig. 1). We have recently reported that cyclic boronates can inhibit representatives of class A, B, and D β-lactamases (21). Cyclic boronates act as analogues of the first tetrahedral intermediate that is common to both SBLs and MBLs (Fig. 1). Here we show that cyclic boronates are able to inhibit all classes of β-lactamase, including the class A ESBL CTX-M-15, the class C enzyme AmpC from Pseudomonas aeruginosa, and two carbapenem-hydrolyzing OXA variants, OXA-23 and OXA-48. These cyclic boronates are effective in inhibiting the growth of clinical Gram-negative bacterial strains expressing multiple β-lactamases. Crystallographic analysis of a cyclic boronate complexed with CTX-M-15 supports the proposal that the cyclic boronates closely mimic the first tetrahedral intermediate in bicyclic β-lactam hydrolysis.

## RESULTS

### Cyclic boronate inhibition of SBLs and MBLs.

To investigate the extent to which cyclic boronates inhibit class C β-lactamases as well as the important class A ESBL and class D carbapenemase targets, we used a fluorogenic assay (22) to screen the cyclic boronates, which we have found to inhibit other β-lactamases (21), against them. The β-lactamases used for screening included TEM-1 and CTX-M-15 (class A), the metallo-β-lactamase (MBL) from Bacillus cereus (BcII), Verona integron-encoded metallo-β-lactamase 1 (VIM-1) (class B), AmpC from P. aeruginosa (class C), and OXA-23 and OXA-48 (class D), collectively representing all classes of β-lactamase. (For comparison with other relevant publications, we also screened CTX-M-15, AmpC, OXA-23, and OXA-48 with the commonly used reporter substrate nitrocefin [23] [see Table S2 in the supplemental material].) To benchmark the potency of the cyclic boronates, we also screened the clinically used serine-β-lactamase (SBL) inhibitors avibactam (MedChemexpress LLC) (16, 24), sulbactam (25, 26), and BLI-489, a potent inhibitor of class D enzymes (2, 27, 28). For MBLs, we used the broad-spectrum thiol-based MBL inhibitors l-captopril (29, 30) and (racemic) thiomandelic acid (31, 32) (Tables 1 and 2) (see Fig. S1 in the supplemental material for structures of the inhibitors). Since variations in the rate of reaction with, at least, avibactam have been reported among the SBLs (16), we also investigated the time courses of inhibition by these compounds over 6 h.

**TABLE 1:** Time course for the inhibition of serine-β-lactamases (classes A, C, and D) by cyclic boronates 1 and 2 and established inhibitors that act by formation of a stable acyl-enzyme complex^*a*^

Inhibitor	Preincubation time (min)	IC_50_, nM (mean ± SD)^*b*^ for:				
		TEM-1	CTX-M-15	AmpC	OXA-23	OXA-48
Cyclic boronate 1	0	2.6 ± 0.1	92 ± 6	68 ± 3	220 ± 1	140 ± 1
	10	1.3 ± 0.1	13 ± 1	9.8 ± 0.3	250 ± 1	160 ± 1
	30	1.6 ± 0.1	3.7 ± 0.1	4.5 ± 0.1	260 ± 1	170 ± 1
	60	1.5 ± 0.1	1.7 ± 0.1	2.6 ± 0.1	270 ± 1	170 ± 1
	360	1.7 ± 0.1	4.0 ± 0.01	2.4 ± 0.1	730 ± 2	270 ± 1
Cyclic boronate 2	0	8.1 ± 0.1	39 ± 2	270 ± 60	2,000 ± 100	2,000 ± 100
	10	3.4 ± 0.1	7.5 ± 0.3	120 ± 10	2,600 ± 100	2,600 ± 200
	30	2.6 ± 0.1	2.8 ± 0.1	150 ± 10	3,300 ± 200	3,400 ± 100
	60	2.6 ± 0.1	1.3 ± 0.1	100 ± 10	2,600 ± 100	3,000 ± 100
	360	2.1 ± 0.1	6.4 ± 0.1	96 ± 1	3,300 ± 200	3,300 ± 200
Sulbactam	0	860 ± 80	44 ± 1	>2 × 10^5^	>2 × 10^5^	>2 × 10^5^
	10	600 ± 200	29 ± 1	42,000 ± 2,000	>2 × 10^5^	>2 × 10^5^
	30	600 ± 200	28 ± 7	8,600 ± 600	>2 × 10^5^	>2 × 10^5^
	60	500 ± 200	32.0 ± 0.3	4,400 ± 500	>2 × 10^5^	>2 × 10^5^
	360	700 ± 300	16.6 ± 0.1	1,000 ± 400	>2 × 10^5^	>2 × 10^5^
Avibactam	0	19 ± 1	9.9 ± 0.2	1,400 ± 400	770 ± 4	2500 ± 30
	10	3.4 ± 0.1	1.1 ± 0.1	190 ± 10	390 ± 1	810 ± 50
	30	2.2 ± 0.1	0.40 ± 0.06	200 ± 10	160 ± 2	300 ± 2
	60	2.0 ± 0.1	0.39 ± 0.01	200 ± 10	71 ± 1	150 ± 1
	360	4.3 ± 0.1	6.4 ± 0.1	150 ± 10	13 ± 1	20 ± 1
BLI-489	0	4.8 ± 0.2	32 ± 2	210 ± 20	5.6 ± 0.1	14 ± 1
	10	2.0 ± 0.1	6.9 ± 0.2	30 ± 1	5.6 ± 0.1	15 ± 1
	30	1.7 ± 0.2	2.2 ± 0.1	12.0 ± 0.4	6.2 ± 0.1	16 ± 1
	60	1.7 ± 0.1	0.94 ± 0.03	5.6 ± 0.1	8.6 ± 0.1	22 ± 1
	360	1.9 ± 0.1	8.0 ± 0.1	1.9 ± 0.1	18 ± 1	49 ± 1

a FC5 was used as a substrate (22).

b IC_50_s were taken after preincubation of the enzyme with the corresponding inhibitor for 0, 10, 30, 60, or 360 min prior to assay. IC_50_s were obtained from fitting of residual activity plots using GraphPad Prism.

**TABLE 2:** Time course for the inhibition of metallo-β-lactamases by cyclic boronates 1 and 2 and broad-spectrum thiol-based MBL inhibitors^*a*^

Inhibitor	Preincubation time (min)	IC_50_, μM (mean ± SD)^*b*^ for:		
		BcII, pH 7.5	BcII, pH 6.5	VIM-1, pH 7.5
1	0	2.8 ± 0.2	3.3 ± 0.1	1 ± 1
	10	3.0 ± 0.2	3.8 ± 0.2	1 ± 1.2
	30	2.8 ± 0.3	3.5 ± 0.1	1 ± 1.5
	60	3.4 ± 0.3	3.8 ± 0.2	1.4 ± 0.3
	360	3.1 ± 0.2	3.0 ± 0.2	1.2 ± 0.3
2	0	0.45 ± 0.02	0.27 ± 0.02	0.061 ± 0.001
	10	0.45 ± 0.02	0.20 ± 0.01	0.085 ± 0.002
	30	0.36 ± 0.02	0.20 ± 0.01	0.088 ± 0.001
	60	0.36 ± 0.01	0.20 ± 0.01	0.083 ± 0.002
	360	0.36 ± 0.03	0.23 ± 0.01	0.061 ± 0.001
l-Captopril	0	13.7 ± 0.3	17.4 ± 0.3	1.91 ± 0.06
	10	21 ± 1	20.4 ± 0.6	2.3 ± 0.2
	30	12.5 ± 0.5	16.8 ± 0.8	2.4 ± 0.2
	60	14.6 ± 0.4	13.7 ± 0.7	2.6 ± 0.1
	360	15 ± 1	16.3 ± 0.5	2.8 ± 0.2
(±)-Thiomandelic acid	0	0.30 ± 0.03	0.27 ± 0.06	0.38 ± 0.03
	10	0.39 ± 0.05	0.9 ± 0.1	0.45 ± 0.02
	30	0.33 ± 0.04	0.5 ± 0.1	0.8 ± 0.9
	60	0.5 ± 0.2	2 ± 1	1.4 ± 0.9
	360	2 ± 1	2 ± 1	2 ± 1

a FC5 was used as a substrate (22).

b IC_50_s were taken after preincubation of the enzyme with the corresponding inhibitor for 0, 10, 30, 60, or 360 min prior to assay. IC_50_s were obtained from fitting of residual activity plots using GraphPad Prism.

Both cyclic boronates 1 and 2 exhibit inhibition against all five of the SBLs tested, with 50% inhibitory concentrations (IC_50_s) ranging from 250 to 2 nM (Table 1). Cyclic boronates 1 and 2 similar inhibition potencies against TEM-1 and CTX-M-15, cyclic boronate 2 shows around 10-fold lower IC_50_s than cyclic boronate 1 against AmpC (9.8 ± 0.3 nM versus 120 ± 10 nM, respectively, with 10 min of preincubation), and cyclic boronate 1 exhibits 10- to 15-fold-lower IC_50_s than cyclic boronate 2 against the OXA enzymes (250 ± 1 nM versus 2,600 ± 100 nM after 10 min of incubation for OXA-23 and 160 ± 1 nM versus 2,600 ± 200 nM after 10 min of incubation for OXA-48). Against TEM-1 and CTX-M-15, cyclic boronates 1 and 2 show potencies similar to those of avibactam and BLI-489, with IC_50_s of low nanomolar to subnanomolar levels. Against AmpC, cyclic boronate 2 shows a potency similar to that of avibactam, while cyclic boronate 1 exhibits up to 60-fold-lower IC_50_s than avibactam (9.8 ± 0.3 nM and 190 ± 10 nM at 10 min, respectively), with results comparable to those for BLI-489 (30 ± 1 nM at 10 min). Neither cyclic boronate 1 nor 2 was able to achieve potency comparable with the lowest IC_50_s exhibited by avibactam and BLI-489 against the OXA enzymes, with IC_50_s for cyclic boronate 1 being around 10- to 20-fold higher and those for cyclic boronate 2 being around 100- to 200-fold higher.

The time dependency of inhibition was found to vary depending on the particular inhibitor-enzyme combination examined (Table 1). Where substantial time dependency in the inhibition was observed, the largest decrease in IC_50_ generally manifested over the first 10 min of inhibition. Avibactam showed time dependency in its inhibition of all the tested SBLs, with the lowest IC_50_ typically achieved after 60 or 360 min of preincubation. The IC_50_s obtained with BLI-489 showed substantial time dependency only with CTX-M-15 and AmpC, with the lowest IC_50_s seen after longer incubation times, as seen with avibactam. The time dependency of inhibition by the cyclic boronates was similar to that observed for BLI-489 (i.e., seen with CTX-M-15 and AmpC but not with TEM-1 or the OXA enzymes) (Table 1). For CTX-M-15, a significant increase in the IC_50_s for avibactam and BLI-489 was observed between 60 and 360 min of incubation. This may be the result of slow hydrolysis of the inhibited acyl-enzyme to restore a small population of active enzyme.

We also screened the cyclic boronates against the model MBL, BcII, and VIM-1 using the FC5-based fluorogenic assay (22). In all cases the inhibition of the MBLs by cyclic boronate 2 was around 10 times more potent than that by cyclic boronate 1 (Table 2). Cyclic boronate 1 exhibited IC_50_s around 2- to 5-fold lower than those seen with l-captopril. Cyclic boronate 2 showed a potency very similar to that of thiomandelic acid against BcII but showed 5- to 10-fold-greater potency than thiomandelic acid against VIM-1. No time dependency of MBL inhibition by either the cyclic boronates or the thiol-based inhibitors was observed (Table 2).

### Susceptibility of clinical isolates to cyclic boronate 2.

Consistent with our previous work (21), cyclic boronate 2 was observed to be a more potent inhibitor of the isolated MBLs than cyclic boronate 1. Since potency against different types of MBLs is a highly desirable characteristic in the design of MBL/SBL dual inhibitors, cyclic boronate 2 (10 μg/ml) was thus tested in combination with a number of β-lactams against a variety of Gram-negative clinical isolates known to produce multiple β-lactamases. Susceptibility to the monobactam aztreonam (AZT) and cephalosporins (ceftriaxone [CRO], ceftazidime [CAZ], and cefepime [FEP]) was either increased in or completely restored to strains producing CTX-M-1-like (CTX-M-15 and CTX-M-27) enzymes. MICs to ampicillin (AMP) and piperacillin (PIP) in combination with fixed ratios of clavulanic acid (CLAV), sulbactam (SUL), and tazobactam (TAZ) were also lower for CTX-M-containing isolates but not significantly lower in those also producing an OXA (OXA-1, OXA-181, or OXA-23) or plasmid-borne AmpC (CMY-4) enzyme. For Enterobacteriaceae producing MBLs, heightened activity was seen with carbapenems against VIM-4-producing Klebsiella pneumoniae and VIM-1-producing Providencia stuartii. There were less marked effects on cephalosporin MICs, presumably due to coproduction of SHV- and VEB-like ESBLs and hyperexpression of chromosomal AmpC in these isolates. In strains with OXA-like carbapenem-hydrolyzing class D β-lactamases (CHDLs), carbapenem susceptibility was increased in Escherichia coli producing the OXA-181 variant, in combination with CTX-M-15 and CMY-4, but not against a multidrug-resistant K. pneumoniae isolate producing the OXA-232 variant in association with CTX-M-15 and multiple other SHV ESBLs. Of note, no significant effects of cyclic boronate 2 on the carbapenem susceptibility of either VIM-2 producing P. aeruginosa or Acinetobacter baumannii with OXA-23 were seen (Table 3).

**TABLE 3:**
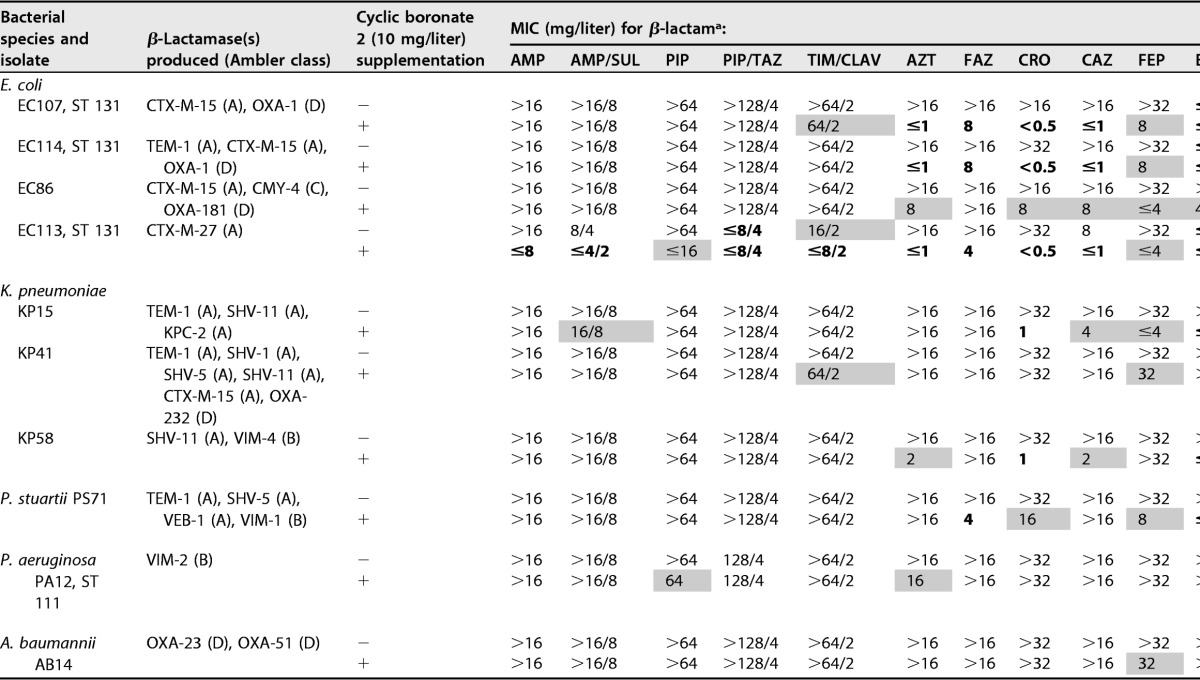
MIC values of selected penicillins, cephalosporins, monobactams, and carbapenems against different bacterial strains with or without cyclic boronate 2 supplementation

^a^MIC values in normal type indicate resistance and those in boldface susceptibility according to current CLSI/EUCAST breakpoints. Shaded values indicate where the MIC is reduced with 10 mg/liter cyclic boronate 2 but either the MIC lies either outside the susceptible range or there is no agreed breakpoint for the drug/organism combination. AMP, ampicillin; AMP/SUL, ampicillin-sulbactam; PIP, piperacillin; PIP/TAZ, piperacillin-tazobactam; TIM/CLAV, ticarcillin/clavulanate; AZT, aztreonam; FAZ, cefazolin; CRO, ceftriaxone; CAZ, ceftazidime; FEP, cefepime; ERT, ertapenem; IMI, imipenem; MEM, meropenem; DOR, doripenem.

Disc diffusion screens in which cyclic boronate 2 was added in a fixed ratio against the same strains revealed some interesting findings on its potential as an inhibitor (see the disc diffusion test images in ths supplemental material). In Enterobacteriaceae, cyclic boronate 2 generally enhanced the activity of PIP, AZT, cefoxitin (FOX), cefotaxime (CTX), and CAZ in those without OXA CHDLs. The effects on carbapenems were clearly observed for the VIM-4-producing strains at the 2:1 ratio.

### ^13^C NMR study with OXA-10.

For the class D OXA enzymes, the active-site lysine (Lys70) is carbamylated via nonenzymatic reaction with carbon dioxide (33). This residue is critical for the activity of the enzymes and acts as a general acid/base during β-lactam hydrolysis (34). Since the carbamylation of Lys70 is reversible, it is possible to site-specifically label the residue with ^13^C, via incubation with a ^13^C-labeled bicarbonate buffer. This labeling allows for changes of the active site (for example, inhibitor binding) to be studied using nuclear magnetic resonance (NMR) (34). Using this reported technique, we labeled the carbamylated Lys70 of OXA-10 with ^13^C (using NaH^13^CO_3_) in order to monitor binding of cyclic boronate 1 within the active site of the enzyme (see Fig. S6 in the supplemental material). Upon binding of the inhibitor, a 6-ppm shift in the ^13^C-labeled carbamylate signal is observed.

### Crystal structure of cyclic boronate 2 bound to CTX-M-15.

Crystal structures of cyclic boronates 1 and 2 in complex with class B (BcII and VIM-2) and D (OXA-10) and PBP-5 from E. coli have been reported (21); however, structural information on inhibition of the clinically important class A β-lactamases, in particular ESBLs, by cyclic boronates has not been described. We thus worked to obtain a structure of the ESBL CTX-M-15:cyclic boronate 1 complex, which diffracted to 1.95-Å resolution (see Table S4 in the supplemental material for crystallographic data). The structure was solved by molecular replacement using the reported structure of the apo-enzyme (PDB accession code 4HBT [35]) as a search model. The overall structure of the CTX-M-15:cyclic boronate 1 complex is highly similar to that of the search model, with a root mean square deviation (RMSD) of 0.194 Å over Cα atoms. In a fashion similar to that seen in a CTX-M-15:avibactam complex crystal structure (PDB accession code 4S2I [36]), comparison with the apo-enzyme reveals no remarkable changes in the positions of the backbone or amino acid side chains upon reaction with cyclic boronate 1.

**FIG 2:**
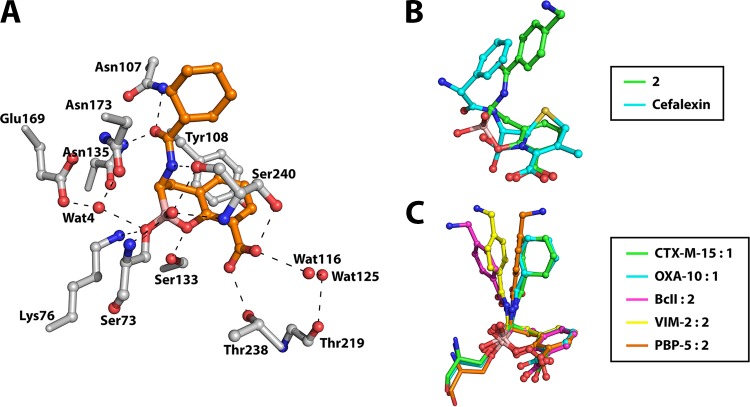
The conformation of the bicyclic ring core of the cyclic boronates in complex with β-lactamases and PBPs is conserved and mimics the tetrahedral anionic intermediate in cephalosporin hydrolysis. (A) Active-site view from a crystal structure of the CTX-M-15:cyclic boronate 1 complex. Potential hydrogen bonding interactions are represented by dashed lines. (B) Overlay of energy-minimized small-molecule structures of cyclic boronate 2 and a modeled species defined by addition of a hydroxide ion onto the β-lactam carbonyl of cefalexin. Energy minimization was carried out using the MM2 energy minimization function in ChemBio3D Ultra. (C) Overlay of our reported (21) cyclic boronate structures in PDB entries 5FQ9 (cyan, OXA-10:cyclic boronate 1), 5FQB (magenta, BcII:cyclic boronate 2), 5FQC (yellow, VIM-2:2), and 5J8X (orange, PBP-5:2) and our CTX-M-15:1 structure (green). Note that there is variation in the conformations of the side chain, but that of the fused bicyclic ring system is highly conserved in all crystal structures and is likely important for optimal binding of the cyclic boronate inhibitors.

Analysis of the electron density maps clearly reveals cyclic boronate 1 as being bound at the active site via reaction with the side chain of Ser73 (Fig. 2A). In a manner analogous to the structures of OXA-10 and PBP-5 with cyclic boronate 2 (21) (PDB accession codes 5FQ9 and 5J8X, respectively), the electron density map provides clear evidence for tetrahedral coordination of the boron atom (see Fig. S7 in the supplemental material). Aside from the covalent reaction with Ser73, cyclic boronate 1 is positioned to form hydrogen bonding interactions with the side chains of Lys76, Asn107, Ser133, Asn135, Thr238, and Ser240 as well as backbone atoms of Ser73 and Ser240 and two nearby water molecules, waters 4 (Wat4) and 116 (Wat116). In addition, there is a hydrophobic/aromatic interaction between the side chain of Tyr108 and the planar aromatic ring of the ligand. Interestingly, and as seen in the CTX-M-15:avibactam complex (36), a water molecule is observed in the CTX-M-15:cyclic boronate 1 complex which occupies the same position as the water responsible for hydrolysis of the acyl-enzyme intermediate in CTX-M-15-catalyzed β-lactam hydrolysis, Wat4 in Fig. 2 and S6 (35).

We then compared the conformation of cyclic boronate 1 in CTX-M-15 with those observed in MBLs (BcII [PDB accession code 5FQB] and VIM-2 [PDB accession code 5FQC]) and OXA-10 (PDB accession code 5FQ9) as well as PBP-5 (PDB accession code 5J8X). Although there are some variations in the precise orientations of the C-7 cyclohexyl amide/aromatic acetamide side chain, the conformation of the fused bicyclic boronate ring system is remarkably well conserved across all the structures analyzed (Fig. 2C). In support of the proposal that the cyclic boronates mimic the first tetrahedral intermediate in β-lactam hydrolysis, which is common in both SBL and MBL catalysis, small-molecule energy minimization studies on cyclic boronate 2 and the tetrahedral species produced by addition of a hydroxide ion onto the β-lactam carbonyl of cefalexin reveal strong structural similarity between the two species.

## DISCUSSION

The results reveal that cyclic boronates 1 and 2, which are based on β-lactamase inhibitors described in patent literature (37), are able to inhibit all classes of β-lactamase, including the important, clinically relevant ESBL CTX-M-15 and the OXA carbapenemases, as well as AmpC from P. aeruginosa, with low micromolar to low nanomolar IC_50_ ranges (Tables 1 and 2). The compounds show inhibition potencies similar to those of avibactam against TEM-1, CTX-M-15, and AmpC. Cyclic boronate 1 was the more potent of the two cyclic boronates against the OXA enzymes but was unable to achieve the potency of avibactam against these enzymes (16). However, the fact that the described cyclic boronate scaffold has yet to be optimized suggests that greater potency for cyclic boronates against the class D β-lactamases should be possible, in line with that seen for other classes. Interestingly, NMR data acquired with OXA-10 show that carbamylation of the active-site lysine is maintained upon binding of cyclic boronate 1 (see Fig. S6 in the supplemental material). The observed shift of 6 ppm is substantial compared to that observed upon binding of hydroxyisopropylpenicillanates, where a shift of 0 to 0.4 ppm is observed (38). The greater shift of the ^13^C signal on binding of the boronate likely reflects the different environment of the active-site serine, being bound to an sp^3^ anionic boron center when complexed to cyclic boronate 1 as opposed to an sp^2^ carbon center in the hydroxyisopropylpenicillanate complex.

Time courses of inhibition against the SBLs reveal that time dependence of IC_50_ is manifest, the magnitude of which is dependent on the enzyme-inhibitor combination employed. This observation is consistent with potential variations in acylation rates seen with avibactam (16) and the SBLs as well as “on-enzyme” fragmentation/cross-linking reactions that can occur after acylation, as demonstrated, for example, in the case of sulbactam (25, 26) and BLI-489 (39). Notably, TEM-1 and CTX-M-15 appear to manifest differences in the time dependency of their inhibition by all five of the tested inhibitors, as has been previously demonstrated for the response of TEM-1 and CTX-M-9 to clavulanic acid, sulbactam, and tazobactam (40), emphasizing the variation in the properties of the β-lactamases even within the same class (5).

Cyclic boronates 1 and 2 exhibit submicromolar IC_50_s against the MBLs BcII and VIM-1, with cyclic boronate 2 being the more potent compound against both enzymes. Inhibition of BcII by cyclic boronate 2 is comparable in potency to that by thiomandelic acid, while cyclic boronate 2 is around 10 times more potent than thiomandelic acid against VIM-1. Avibactam is a potent inhibitor of SBLs but, notably, is hydrolyzed slowly by some MBLs (19, 41). The time courses of cyclic boronate inhibition against the enzymes tested demonstrated no time dependence for inhibition of the MBLs, at least under our experimental conditions. Despite avibactam being a potent inhibitor of SBLs, it is able to be hydrolyzed by MBLs (19), suggesting potential problems in the long-term clinical use of avibactam (and other inhibitors working by acylation) as resistance mediated by combinations of SBLs and MBLs become more prevalent (20). In contrast, no β-lactamase-catalyzed turnover of the cyclic boronates has been seen (as expected), and, to date, we have no evidence for these compounds binding to β-lactamases or PBPs in a ring-opened fashion. This is consistent with a recent publication suggesting that, at least for 6-member boronate rings, the closed form is the dominant species in solution (42).

Cyclic boronate 2 potentiated the activity of all four classes of β-lactam against Gram-negative clinical isolates (Table 3). Coadministration of cyclic boronate 2 alongside the clinically used SBL inhibitors clavulanic acid, sulbactam, and tazobactam was able to further potentiate the activity of penicillins against some E. coli strains (EC107 and EC113) compared to coadministration with the SBL inhibitors alone. Cyclic boronate 2 also increased the effectiveness of both cephalosporins and carbapenems against VIM-producing K. pneumoniae strains, although this result is not apparent with KP41, possibly due to the sheer number of β-lactamases (six) being produced. Activity against the VIM-2-producing P. aeruginosa strain was also limited. Mechanisms of multidrug resistance in this strain have not yet been fully elucidated, although production of the native AmpC β-lactamase combined with upregulated efflux and permeability lesions is likely to be involved. An increased susceptibility to ceftolozane was a common finding regardless of the number or class of β-lactamase produced, except in A. baumannii. Ceftolozane is a fifth-generation cephalosporin, recently developed for use in combination with tazobactam (43). The limited isolate results presented here suggest that ceftolozane, partnered with a cyclic boronate, could be an attractive β-lactam/inhibitor combination to pursue in the future.

A structure of the CTX-M-15:cyclic boronate 1 complex reveals a conserved mode of binding for this inhibitor, very similar to our previously reported structures, with a tetrahedral boron center and the closed bicyclic scaffold maintained as in our previously reported work (21). Comparison of our structure with that of CTX-M-15 in complex with RPX-7009, a boron-based SBL inhibitor currently in phase III clinical trials (44, 45), and a TEM-1:boronate (46) complex reveals a striking similarity in the mode of binding for these related scaffolds (see Fig. S8 in the supplemental material). Interestingly, the latter structure was interpreted as an acyclic boronate binding mode, despite the compound being able to adopt a bicyclic conformation nearly identical to that of our inhibitors via reaction of its phenolic oxygen with the boron center (Fig. S8C) (46). It should be noted that, in contrast to the structural conservation observed for the cyclic boronate complex conformations, there is considerably more variation in the structural conformations of acyl-enzyme (and product) complexes formed by the reaction of SBLs with β-lactams and of reported product/intermediate complexes formed from MBLs and β-lactams (see Fig. S9 in the supplemental material) (34, 47–49). Since more conformational flexibility might be anticipated once the β-lactam ring has been opened, this analysis supports the proposal that the cyclic boronates best mimic the first tetrahedral intermediate (i.e., act as “transition state” analogues).

The cyclic boronate scaffold is thus able to potently inhibit all classes of β-lactamase by adopting an enzyme:inhibitor complex that mimics a tetrahedral intermediate in β-lactam hydrolysis. With further optimization, cyclic boronates could form a new family of clinically useful β-lactamase, and maybe other hydrolytic enzyme, inhibitors. In addition to their inhibitory properties, the cyclic boronates also provide important structural and mechanistic insights into the nature of β-lactamase:β-lactam complexes, allowing us to build a more detailed picture of more transient species that have yet to be structurally characterized.

With the first cyclic boronate drug, tavaborole (50), approved for clinical use in the treatment of external fungal infections and further cyclic boronates in the pipeline as anti-inflammatory and anti-bacterial treatments (51, 52), the use of cyclic boronates as future drug candidates seems to be an inevitability. The ability of these molecules to mimic tetrahedral intermediates in enzyme-catalyzed hydrolysis pathways may prove to be highly useful in the inhibition of mechanistically diverse enzymes, as exhibited by the ability of cyclic boronates 1 and 2 to inhibit all classes of β-lactamase, including serine-, metallo-, or other enzymes. In addition, similar classes of compounds, such as cyclic phosphonates, sulfonates, and sulfonamides, have yet to be extensively explored and may prove to be a fruitful source of future inhibitors.

## MATERIALS AND METHODS

### Cloning.

Serine β-lactamases were amplified by direct PCR from producer bacterial strains and expressed as N-terminal hexahistidine fusions from the T7 vector pOPINF (53). CTX-M-15 was amplified from E. coli strain EO516 (a kind gift from Neil Woodford, Public Health England) (54), P. aeruginosa AmpC was amplified from strain PAO1 (a kind gift from the former Pseudomonas genetic stock center, East Carolina University) (55), and OXA-23 and OXA-48 were amplified from clinical Acinetobacter baumannii and Klebsiella pneumoniae isolates (kind gifts from Timothy Walsh and Mark Toleman, Cardiff University). β-Lactamase open reading frames encoding the mature polypeptides (i.e., with regions encoding the signal peptide removed) were amplified by PCR using Phusion polymerase (New England BioLabs) and primers as detailed in Table S1 in the supplemental material. PCR products were cloned into the pOPINF T7 expression vector (53), linearized at the KpnI and HindIII sites using the InFusion recombinase system (Clontech) (56), and transformed into E. coli Stellar (Clontech), and positive clones were selected by blue-white screening. Recombinant plasmids were purified and sequenced (Eurofins Genomics) to confirm identity with published sequences and that no mutations had been introduced during the cloning procedure. The resulting expression constructs encode (exclusive of vector-derived amino acid residues) mature β-lactamase sequences starting at residues 29 (CTX-M-15), 27 (AmpC), 18 (OXA-23), and 22 (OXA-48).

### Enzyme production.

Recombinant CTX-M-15, AmpC, OXA-23, and OXA-48, each with an N-terminal His tag, were produced in E. coli BL21(DE3) cells using autoinduction medium supplemented with 50 µg/ml ampicillin. Cells were grown for 4 h at 37°C before cooling to 18°C and continuing growth overnight. Cells were harvested by centrifugation (10 min, 10,000 × *g*), resuspended in 50 ml lysis buffer (50 mM HEPES [pH 7.5], 500 mM NaCl, 5 mM imidazole), supplemented with DNase I, and lysed by sonication. The supernatant was loaded onto a 5-ml HisTrap HP column, followed by extensive washing with 50 mM HEPES (pH 7.5)–500 mM NaCl–5 mM imidazole before elution with a 20 to 500 mM imidazole gradient. Fractions containing purified enzyme were concentrated by centrifugal ultrafiltration (Amicon Ultra [Millipore]; 15 ml, 10,000 molecular weight cutoff). The resultant solution was injected onto a Superdex S200 column (300 ml) and eluted with 50 mM HEPES (pH 7.5)–200 mM NaCl. Fractions containing pure His-tagged enzyme were incubated overnight at 4°C with His-tagged 3C protease (1:100, wt/wt) to remove the N-terminal His tag. The 3C protease together with any uncleaved protein in the digestion mixture was removed by use of a second HisTrap HP column preequilibrated with 50 mM HEPES (pH 7.5)–500 mM NaCl–20 mM imidazole. Purified enzyme fractions, as identified by SDS-PAGE, were pooled and concentrated by centrifugal ultrafiltration before buffer exchange into 25 mM HEPES (pH 7.5)–100 mM NaCl. The concentrations of the purified proteins were determined using a NanoDrop ND-1000 spectrophotometer (Thermo Scientific; ε = 25,440, 61,310, 43,430, and 63,940 M^−1^ cm^−1^ for CTX-M-15, AmpC, OXA-23, and OXA-48, respectively).

Recombinant TEM-1 (57) with an N-terminal His tag, VIM-1 (58) with a 3C-cleaved C-terminal His tag, and OXA-10 (59) were produced as previously described. The concentrations of the purified proteins were determined using a NanoDrop ND-1000 spectrophotometer (Thermo Scientific; (ε = 27,960, 29,910, and 48,930 M^−1^ cm^−1^ for TEM-1, VIM-1, and OXA-10, respectively).

### ^13^C labeling of OXA-10 enzyme.

The ^13^C labeling of carbamylated lysine was based on a protocol in the literature (34). Purified OXA-10 enzyme was dialyzed first overnight against degassed 25 mM sodium acetate (pH 4.5)–0.1 mM EDTA and then overnight against 50 mM sodium phosphate (pH 7.4)–0.1 mM EDTA–1 mM NaH^13^CO_3_ (Sigma-Aldrich). The enzyme was then dialyzed overnight against 50 mM sodium phosphate (pH 7.4)–0.1 mM EDTA–10 mM NaH^13^CO_3_, aliquoted, and frozen using liquid N_2_.

### Inhibition assays.

Inhibition assays were carried out using FC5 as a fluorogenic reporter substrate (22). Enzyme concentrations and buffers were the same as those employed in steady-state kinetic studies (see Table S2 in the supplemental material). TEM-1, BcII, and VIM-1 were screened at 1 nM, 500 pM, and 125 pM, respectively. The concentration of FC5 employed was 10 μM for TEM-1 and 5 μM for all other enzymes. IC_50_s were determined by preincubating the enzyme with the inhibitor in the assay buffer at room temperature for 10, 30, 60, or 300 min prior to the addition of substrate. Data at 0 min of incubation were obtained by addition of enzyme to a premixed solution of inhibitor and substrate. Residual enzyme activity was determined for a range of inhibitor concentrations. Nonlinear regression fitting of IC_50_ curves was carried out using a three-parameter dose-response curve in GraphPad Prism. Errors in IC_50_ are expressed as [σ(log IC_50_)/log IC_50_)] × IC_50_.

### Antimicrobial susceptibility testing.

The *in vitro* activity of cyclic boronate 2 was assessed using nine clinical isolates carrying multiple β-lactamases (Table 3). The isolates selected included Enterobacteriaceae (E. coli ST 131, Klebsiella pneumoniae ST 258, and Providencia stuartii) producing class A ESBLs (CTX-M-15, CTX-M-27, SHV-5, and VEB-1), serine carbapenemase (KPC-2) and metallo-carbapenemases (VIM-1 and VIM-2), plasmid-mediated AmpC (CMY-2), and/or carbapenem-hydrolyzing OXA-48-like oxacillinases (OXA-181 and OXA-232) in various combinations. The activity against carbapenemase-producing strains of Pseudomonas aeruginosa (VIM-2) and Acinetobacter baumannii (OXA-23) was also investigated. All isolates have previously undergone extensive phenotypic and genotypic characterization (60).

Bacterial susceptibility to β-lactams and standard β-lactam inhibitor (clavulanic acid [CLAV], sulbactam [SUL], and tazobactam [TAZ]) combinations was determined by broth microtiter dilution (BMD) according to the Clinical and Laboratory Standards Institute (CLSI) methodology (61). MICs were determined using commercial Sensititre GN4F panels (Thermo Scientific, UK; lot B55051) and Mueller-Hinton II cation-adjusted broth (Oxoid, UK), with and without the addition of cyclic boronate 2 at a fixed concentration of 10 μg/ml. Plates were incubated at 37°C for 18 h and MICs read by eye following the addition of alamarBlue reagent (Trek Diagnostics).

The inhibitory effects of cyclic boronate 2 on susceptibility to 19 diverse β-lactam compounds was also assessed in Kirby-Bauer disc diffusion tests. Combination discs (Oxoid) were prepared with a fixed ratio of 2:1 between the β-lactam (μg) and cyclic boronate 2. Zones of inhibition around combined and unsupplemented discs were compared following overnight incubation on MH II plates.

### NMR spectroscopy.

^13^C NMR experiments used a Bruker AVIII 600 MHz spectrometer equipped with a Prodigy broadband cryoprobe. Spectra were acquired at 298 K using a standard Bruker ^13^C pulse sequence. The experimental parameters used were as follows: 2,048 scans, 36,058-Hz spectral width, 2.0-s relaxation delay, and 65,536 data points. A line broadening of 10 Hz was applied to all spectra. NMR samples contained 560 μM OXA-10 and 10 mM NaH^13^CO_3_ and were supplemented with 10% D_2_O. The impact of cyclic boronate 1 was tested at a concentration of 5 mM.

### Crystallization, X-ray data collection, and processing.

Crystallization experiments were set up using a 13 mg/ml solution of CTX-M-15 in 50 mM HEPES (pH 7.5)–100 mM NaCl supplemented with 10 mM cyclic boronate 1. Crystallization was performed at room temperature using sitting-drop vapor diffusion methods. Crystals were obtained after 2 days using 100 μl of 100 mM HEPES (pH 7.5)–70% 2,4-methylpentanediol in the reservoir and a 1:2 mixture (1 μl:2 μl) of protein to reservoir solution in the crystallization drop. Crystals were cryoprotected using 25% glycerol in reservoir solution before harvesting with nylon loops and flash-cooling in liquid nitrogen. Diffraction data were collected at 100 K using a Rigaku FRE+ Superbright diffractometer. Diffraction data were integrated and scaled using HKL3000 (62). The structure was solved by molecular replacement with Phaser (63) using a published structure (PDB accession code 4HBT [35]) as a search model. The structure was then fit and refined iteratively using PHENIX and Coot (64, 65).

### Structural energy minimization.

Energy minimization of small-molecule structures was performed using the MM2 energy minimization function in ChemBio3D Ultra (66).

### Accession number(s).

Coordinates and structure factors have been deposited in the Protein Data Bank with accession number 5T66.

## Supplementary Material

Supplemental material
