# Supplementary material for: Cyclic Boronates Inhibit All Classes of β-Lactamases
Source: Antimicrob Agents Chemother. 2017 Mar 24;61(4):e02260-16. doi: 10.1128/AAC.02260-16 (PMC5365654; doi:10.1128/AAC.02260-16)
Supplement: Supplemental material [file supp_61_4_e02260-16__index.html]

Supplemental material 

# Cyclic Boronates Inhibit All Classes of β-Lactamases

## Supplemental material

- Supplemental file 1 -

  Supplemental text, Tables S1 to S4, and Fig. S1 to S9

  PDF, 1.2M
