# Supplementary material for: Cyclic Boronates Inhibit All Classes of β-Lactamases
Source: Antimicrob Agents Chemother. 2017 Mar 24;61(4):e02260-16. doi: 10.1128/AAC.02260-16 (PMC5365654; doi:10.1128/AAC.02260-16)
Supplement: Supplemental material [file AAC.02260-16_zac004176027s1.pdf]

Cyclic Boronates Inhibit All Classes of β-Lactamase

Samuel T. Cahill[a], Ricky Cain[d], David Y. Wang[a], Christopher T. Lohans[a], David W. Wareham[b], Henry P. Oswin[c], Jabril Mohammed[c], James Spencer[c], Colin W. G. Fishwick[d], Michael A. McDonough[a], Christopher J. Schofield[a†] & Jürgen Brem[a†]

[a]Chemistry Research Laboratory, University of Oxford, Oxford, UK

[b]Queen Mary University of London, London, UK

[c]School of Cellular and Molecular Medicine, University of Bristol, Bristol, UK

[d]School Of Chemistry, University of Leeds, Leeds, UK

[†]Corresponding author (Jürgen Brem email: jurgen.brem@chem.ox.ac.uk, Christopher Schofield email: christopher.schofield@chem.ox.ac.uk)

Supplemental Material

**Supplemental Methods**	**Page 3**
**Supplemental Tables**	**Pages 4 - 7**
**Supplemental Figures**	**Pages 8 - 15**
**Supplemental Disc Diffusion Test Images**	**Pages 16 – 26**
**References**	**Page 27**

Supplemental Methods

Steady-state kinetics

Steady-state kinetics studies used a Pherastar FS microplate reader with UV-STAR® 96-well, half area, µclear® plates (Greiner Bio-One) for nitrocefin experiments and 96-well, half area, black, µclear® plates (Greiner Bio-One) for FC5 (1). Buffers employed were 100 mM phosphate buffer pH 7.5, 0.01% (v/v) Triton X-100 for TEM-1, CTX-M-15 and AmpC, 100 mM phosphate buffer pH 7.5, 50 mM sodium bicarbonate, 0.01% Triton X-100 for OXA-23 and OXA-48 and 50 mM HEPES pH 7.5, 1 µM $ZnSO_4$, 0.01% Triton X-100 and 50 mM MES pH 6.5, 1 µM $ZnSO_4$, 0.01% Triton X-100 for MBL assays. Reaction progress was monitored at 298 K by following changes in absorbance at 485 nm for nitrocefin and changes in fluorescence intensity (λ_{ex} = 380 nm, λ_{em} = 480 nm) for FC5 (1). Suitable enzyme concentrations for kinetic analysis were determined by monitoring changes in absorbance/fluorescence for enzyme concentrations between 20 nM and 20 pM. The determined values are provided in Table S2. Kinetic constants were determined using the initial rate of hydrolysis with the reaction initiated through the addition of enzyme to pre-prepared substrate concentrations. Substrate concentrations ranged between 400 µM and 0.4 µM. The Michaelis-Menten equation was fitted to data by non-linear regression using GraphPad Prism software to calculate the Michaelis-Menten constant and the limiting rate (2).

Supplemental Tables

Primer Name	Sequence
CTXM15pOPINFFwd	5'-AAGTTCTGTTTCAGGGCCCGCAAACGGCGGACGTAC-3'
CTXM15pOPINFRev	5'-ATGGTCTAGAAAGCTTTACAAACCGTCGGTGAC-3'
PAAmpCpOPINFFwd	5'-AAGTTCTGTTTCAGGGCCCGGGCGAGGCCCCGGC-3'
PAAmpCpOPINFRev	5'-ATGGTCTAGAAAGCTTTATCAGCGCTTCAGCGGCACC-3'
OXA23pOPINFFwd	5'-AAGTTCTGTTTCAGGGCCCGTGTACGGTTCAGCATAATTTAATAAA-3’
OXA23pOPINFRev	5'-ATGGTCTAGAAAGCTTTAAATAATATTCAGCTGTTTTAATGATTTC-3’
OXA48pOPINFFwd	5'-AAGTTCTGTTTCAGGGCCCGAAGGAATGGCAAGAAAACAAAAG-3'
OXA48pOPINFRev	5'-ATGGTCTAGAAAGCTTTAGGGAATAATTTTTTCCTGTTTG-3'

Table S1. Sequences of primers used in the cloning of CTX-M-15, *Pseudomonas aeruginosa* AmpC, OXA-23 and OXA-48.

Enzyme	Substrate	[E] (pM)	k_{cat} (s^{-1})	K_m (μM)	k_{cat}/K_m (μM s^{-1})
CTX-M-15	Nitrocefin*	100	1000 ± 120	80 ± 14	13
	FC5	50	1100 ± 100	170 ± 20	6.5
AmpC	Nitrocefin	250	540 ± 40	100 ± 20	5.4
	FC5	500	500 ± 160	600 ± 220	0.83
OXA-23	Nitrocefin	2000	2800 ± 200	50 ± 9	56
	FC5	1000	38 ± 7	500 ± 130	0.076
OXA-48	Nitrocefin	2000	4800 ± 200	62 ± 8	77
	FC5	1000	28 ± 2	420 ± 50	0.067

Table S2. Kinetic constants for the β-lactamase-catalysed hydrolysis of FC5 (1) and, for comparison the more commonly used reporter substrate nitrocefin (3). Data were fitted using GraphPad Prism 5 (2). *Indicates that substrate-based inhibition was observed within the concentration range employed in the experiment.

Isolate	β-Lactamases Produced	Synergy When Combined with (2): Fixed 2:1 µg Ratio																		
		CAR	PRL	TZP	KF	C/T	TEM	MEL	CTX	CAZ	SAM	CPT	FEP	AZT	IPM	CAZ/AVI	AUG	ERT	FOX	MEM
E.coli (EC107) ST 131	CTX-M-15 (A), OXA-1 (D)	N	Y	N	N	N	Y	N	Y	Y	N	Y	Y	Y	N	N	N	N	Y	N
E.coli (EC114) ST 131	TEM-1 (A), CTX-M-15 (A), OXA-1 (D)	N	Y	N	N	N	Y	N	Y	Y	N	Y	Y	Y	N	N	N	N	Y	N
E.coli (EC86)	CTX-M-15 (A), CMY-4 (C), OXA-181 (D)	N	N	Y	N	Y	N	N	N	N	N	N	N	Y	N	N	N	N	N	N
E.coli (EC113) ST 131	CTX-M-27 (A)	N	Y	N	Y	N	N	N	Y	Y	N	Y	Y	Y	N	N	Y	N	N	N
K. pneumoniae (KP15)	TEM-1 (A), SHV-11 (A), KPC-2 (A)	N	Y	Y	N	Y	Y	Y	Y	N	N	N	Y	Y	Y	N	N	Y	Y	Y
K. pneumoniae (KP41)	TEM-1 (A), SHV-1 (A), -5 (A), -11 (A), CTX-M-15 (A), OXA-232 (D)	N	N	N	N	Y	N	N	N	N	N	N	N	N	N	N	N	N	N	N
K. pneumoniae (KP58)	SHV-11 (A), VIM-4 (B)	N	Y	Y	N	Y	Y	Y	Y	N	N	N	Y	Y	Y	N	N	Y	N	Y
P. stuartii (PS71)	TEM-1 (A), SHV-5 (A), VEB-1 (A), VIM-1 (B)	N	Y	N	N	Y	N	N	Y	N	N	N	Y	Y	N	N	N	N	N	N
P. aeruginosa (PA12) ST 111	VIM-2 (B)	N	Y	Y	N	Y	N	N	N	N	N	N	N	Y	N	N	N	N	N	N
A. baumannii (AB14)	OXA-51 (D), OXA-23 (D)	N	N	N	N	N	N	N	N	N	N	N	Y	N	N	N	N	N	N	N

Supplementary Table S3: Synergy in Disc Diffusion Assays between 2 and β-lactams: CAR: Carbenacillin; PRL: Piperacillin; TZP: Piperacillin/Tazobactam; KF: Cephalothin; C/T: Ceftolozane/Tazobactam; TEM: Temocillin; MEL: Mecillinam; CTX: Cefotaxime; CAZ: Ceftazidime; SAM: Ampicillin/Sulbactam; CPT: Ceftaroline; FEP: Cefepime; AZT: Aztreonam: IPM: Imipenem; CAZ/AVI: Ceftazidime/Avibactam; AUG: Amoxicillin/Clavulanate; ERT: Ertapenem; FOX: Cefoxitin; MEM: Meropenem.

Data Set	CTX-M-15:1 Complex
Data Collection	
Source	**Rotating Copper Anode**
Resolution Range (Å)	**21.68 – 1.95 (2.02 – 1.95)[a]**
Space Group	**$P\ 2\ 2_1\ 2_1$**
Unit Cell Parameters	
a, b, c (Å)	**43.5, 76.1, 149.4**
α, β, γ (°)	**90.0, 90.0, 90.0**
Unique Reflections	**36639 (3565)[a]**
Completeness (%)	**99.07 (99.56)[a]**
Redundancy	**5.3**
R_{merge}	**0.12 (0.10)[a]**
<I/σ(I)>	**5.3 (1.6)[a]**
Refinement	
R_{work}/R_{free}	**0.2003/0.2341**
RMSD	
Bonds (Å)	**0.003**
Angles (°)	**0.61**
Average B-factor for protein atoms (Å^2)	**19.31**
Ramachandran Plot	
Most Favoured Geometry (%)	**98.0**
Additionally Allowed (%)	**1.1**
Outliers (%)	**0.4**

Table S4: Crystallographic data and refinement statistics for the CTX-M-15:1 complex. [a]Values for the highest resolution shell.

Supplemental Figures

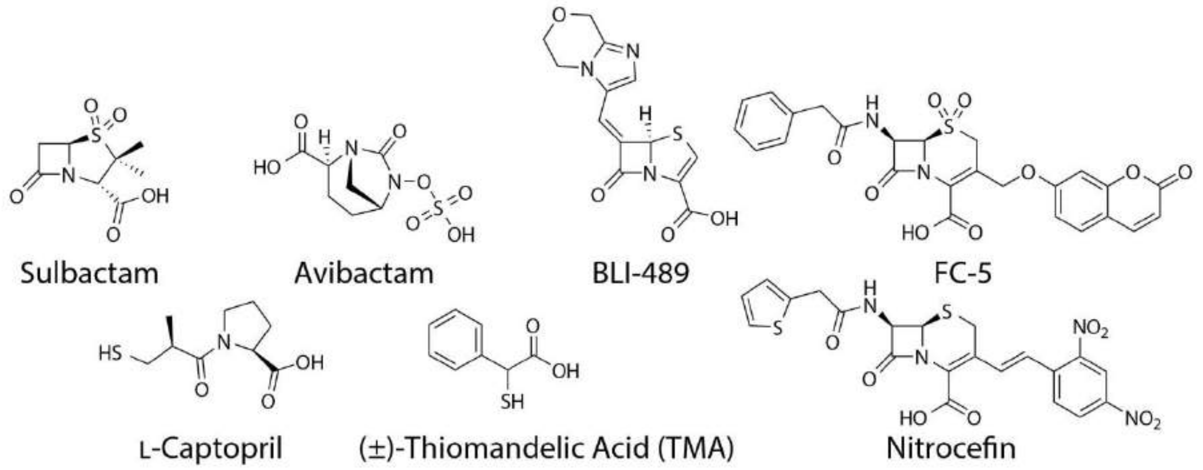

Figure S1: Structures of substrates and inhibitors used in this work.

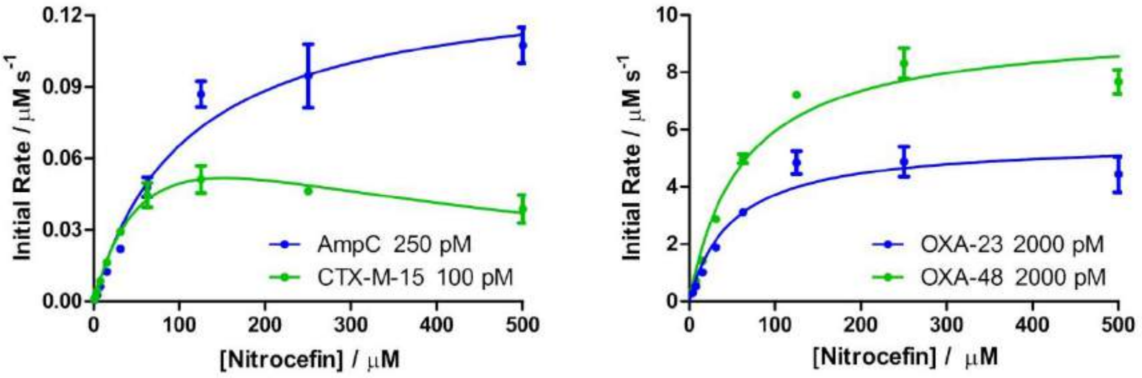

Figure S2: Hydrolysis of nitrocefin catalysed by CTX-M-15, AmpC, OXA-23 and OXA-48. The initial rate of reaction is plotted against substrate concentration. The concentrations of the enzymes employed in the work are indicated. Solid lines indicate the Michaelis-Menten curve fitted using GraphPad Prism.

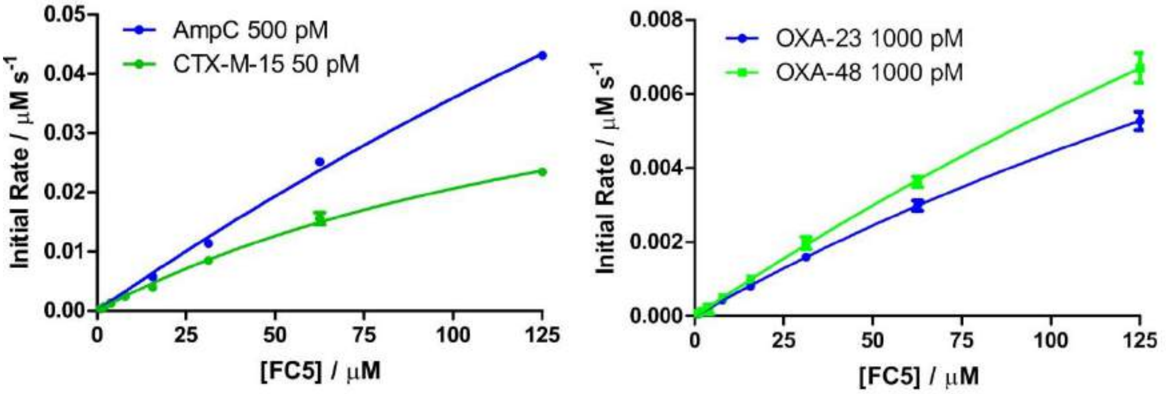

Figure S3: Hydrolysis of FC5 catalysed by CTX-M-15, AmpC, OXA-23 and OXA-48. The initial rate of reaction is plotted against substrate concentration. The concentrations of the enzymes employed in the work are indicated. Solid lines indicate the Michaelis-Menten curve fitted using GraphPad Prism.

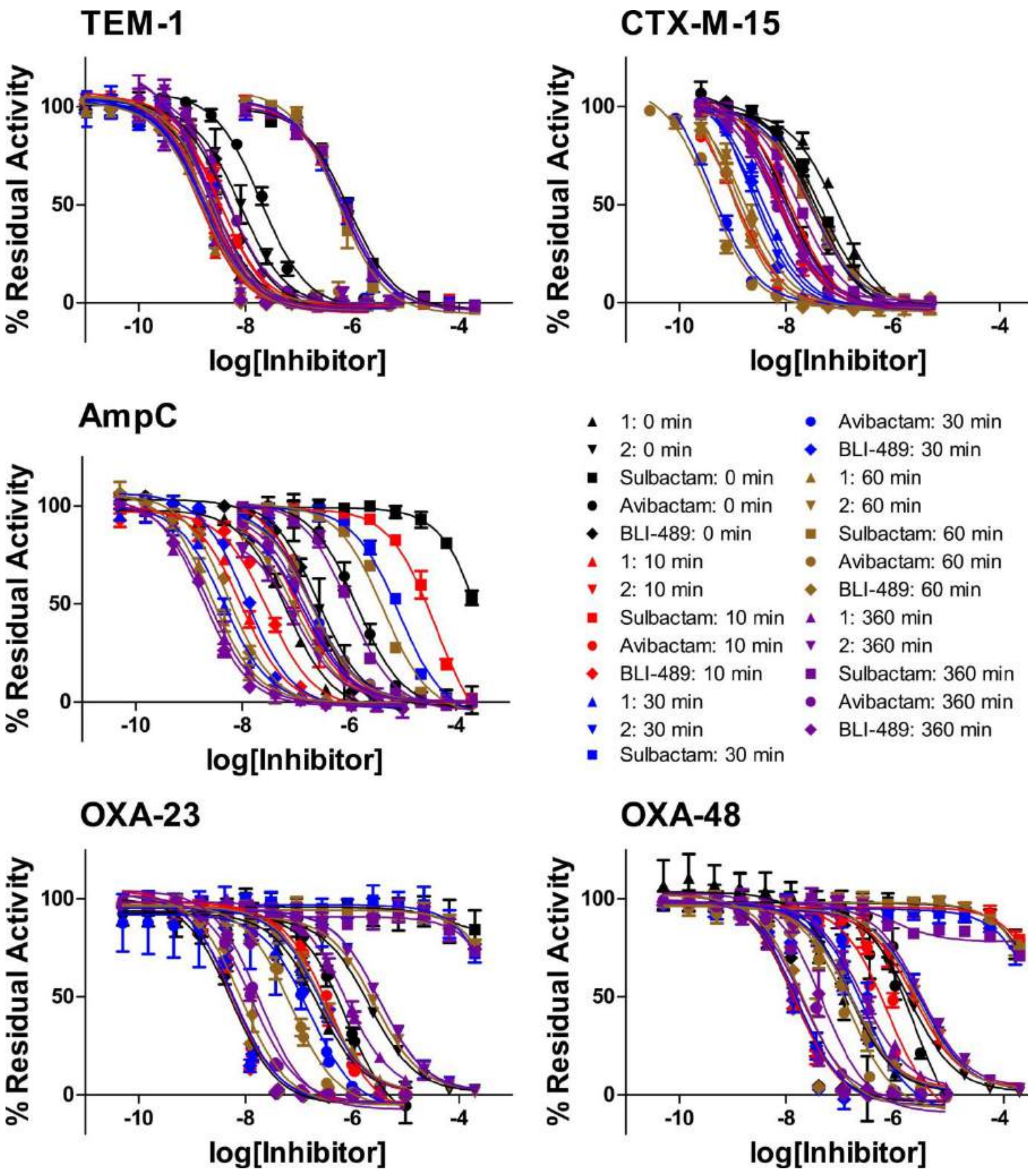

Figure S4: IC_{50} curves for serine-β-lactamase inhibition time courses. Residual activities were calculated from the initial rate of FC5 hydrolysis after incubation of the enzyme with the inhibitor for 0, 10, 30, 60 or 360 minutes.

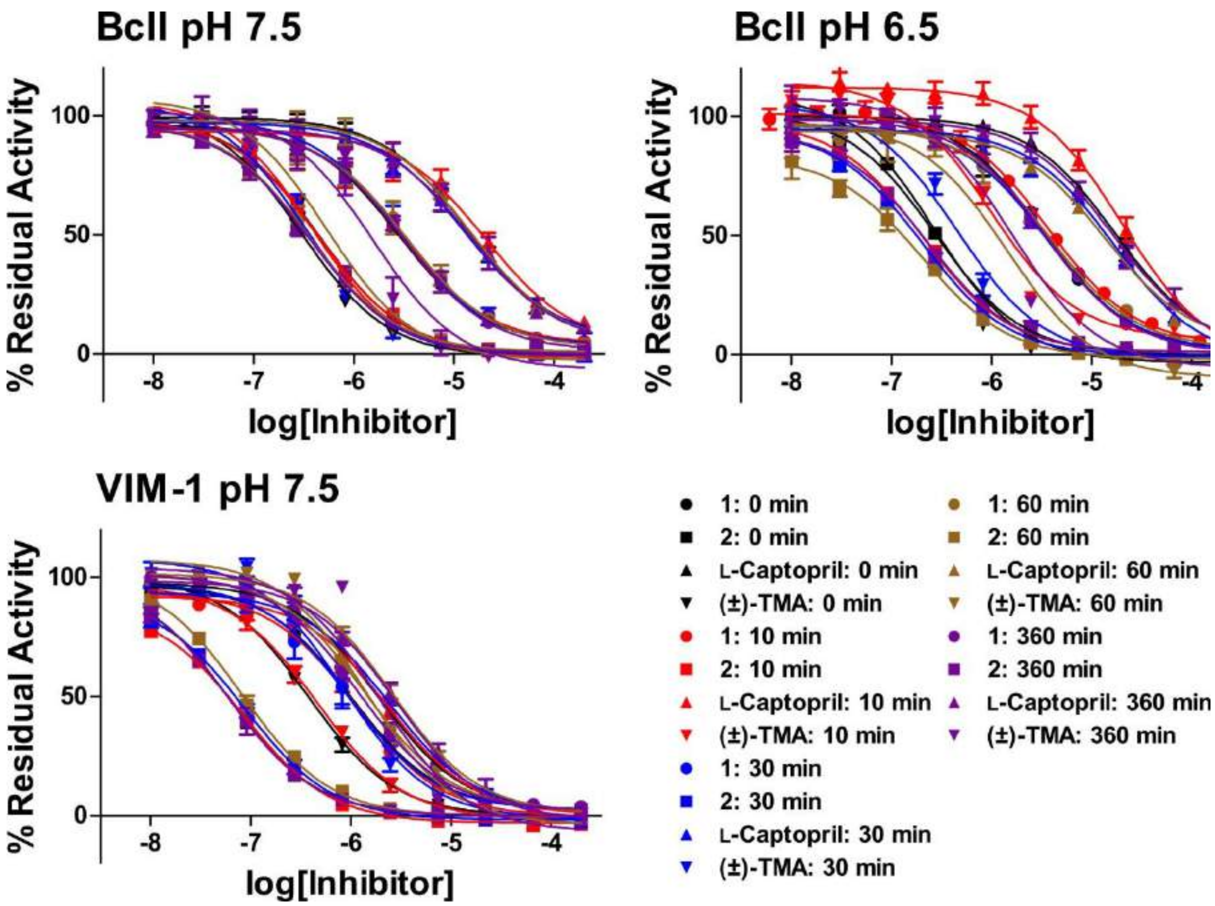

Figure S5: IC$_{50}$ curves for metallo-β-lactamase inhibition time courses. Residual activities were calculated from the initial rate of FC5 hydrolysis after incubation of the enzyme with the inhibitor for 0, 10, 30, 60 or 360 minutes.

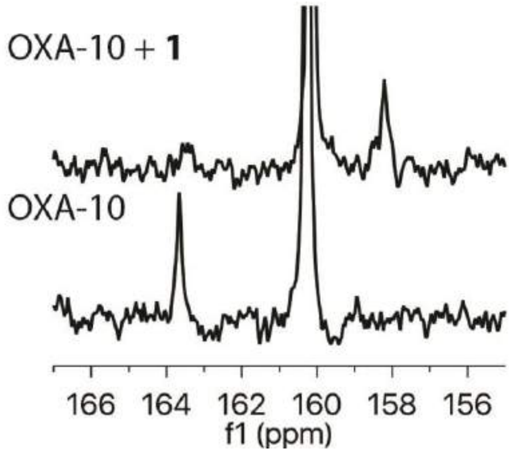

Figure S6. Impact of boronate 1 on carbamylation of the OXA-10 active site lysine residue. ^{13}C NMR reveals a change in the environment of the carbamylated lysine upon binding of 1, with a corresponding shift of 6 ppm. Notably, the carbamylation state of the lysine is maintained on inhibitor binding.

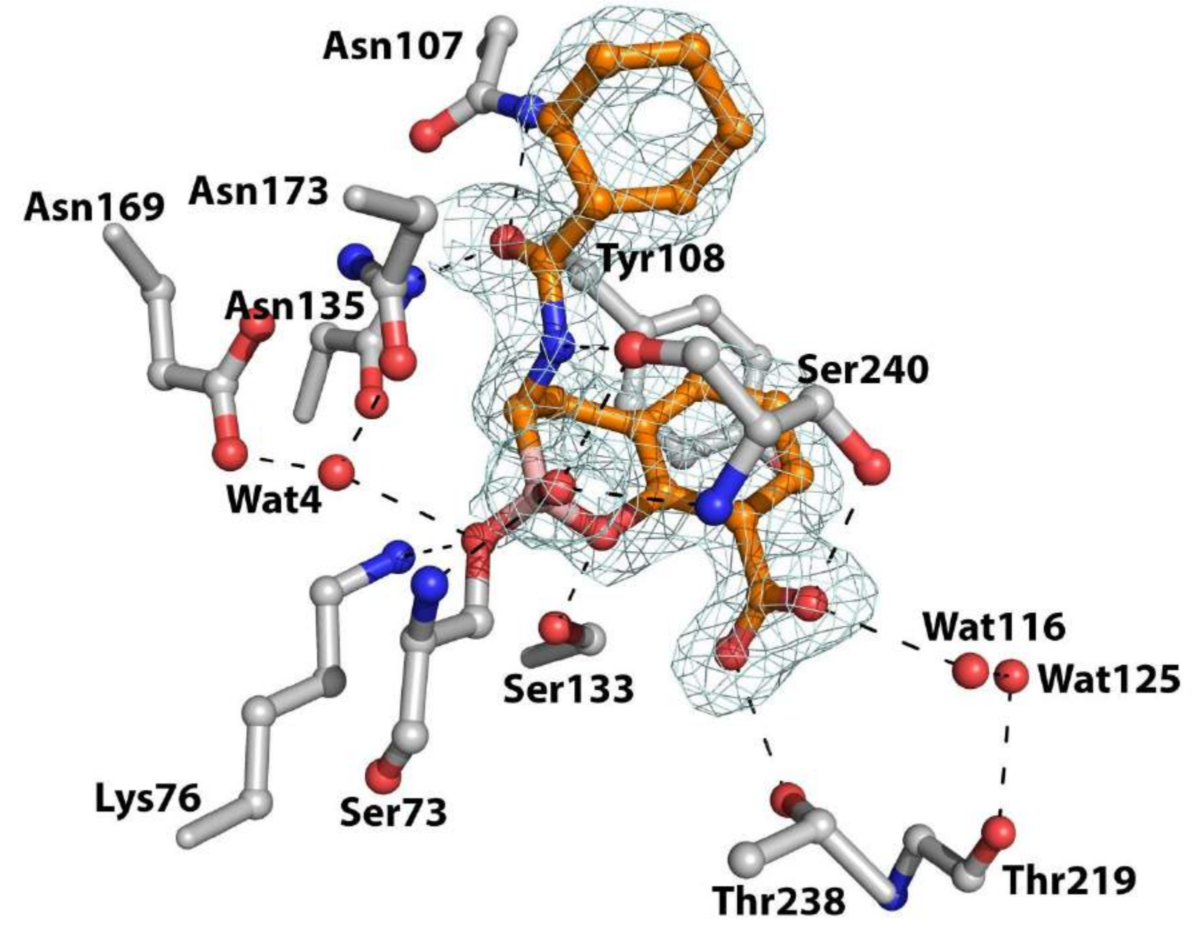

Figure S7: View from a crystal structure of the CTX-M-15 active site with bound cyclic boronate 1, PDB accession code: 5T66. Representative electron density for 2 is shown (3.0 σ $_{m}F_{o}$-DF_{c} OMIT, grey mesh).

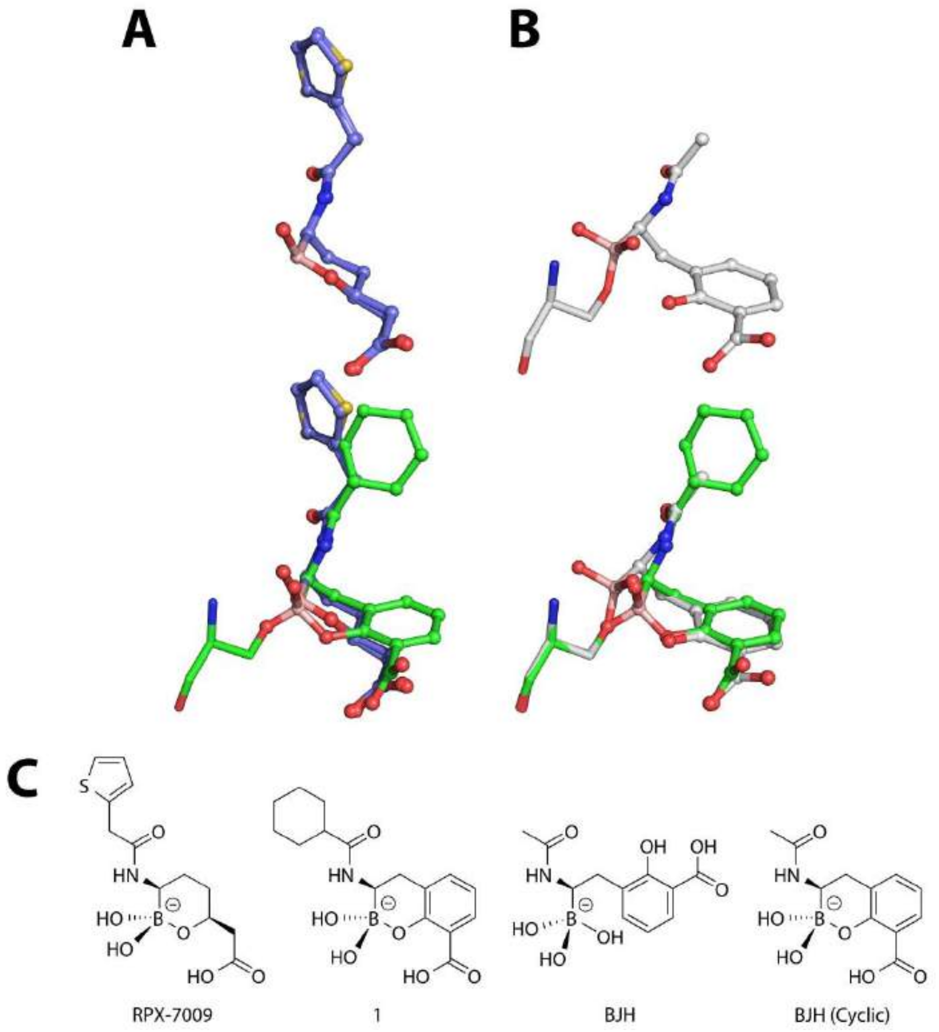

Figure S8. A. The conformation of the monocyclic boronate inhibitor RPX-7009 when bound to CTX-M-15 (blue, PDB accession code: 4XUZ (4)) and an overlay with that of 1 as observed in the CTX-M-15:1 structure (green, PDB Accession Code: 5T66). B. The conformation of the inhibitor BJH when bound to TEM-1 (grey, PDB Accession Code: 1ERQ (5)) and an overlay with that of 1 as observed in the CTX-M-15:1 structure (green, PDB Accession Code: 5T66). Note that BJH has the potential to form a bicyclic scaffold in the same manner as 2 (via formation of a bond between its phenolic oxygen and the boron, BJH (Cyclic)), but the boronate is interpreted as acyclic in the deposited complex structure. C. The chemical structures of RPX-7009, 1 and BJH in open and cyclic forms.

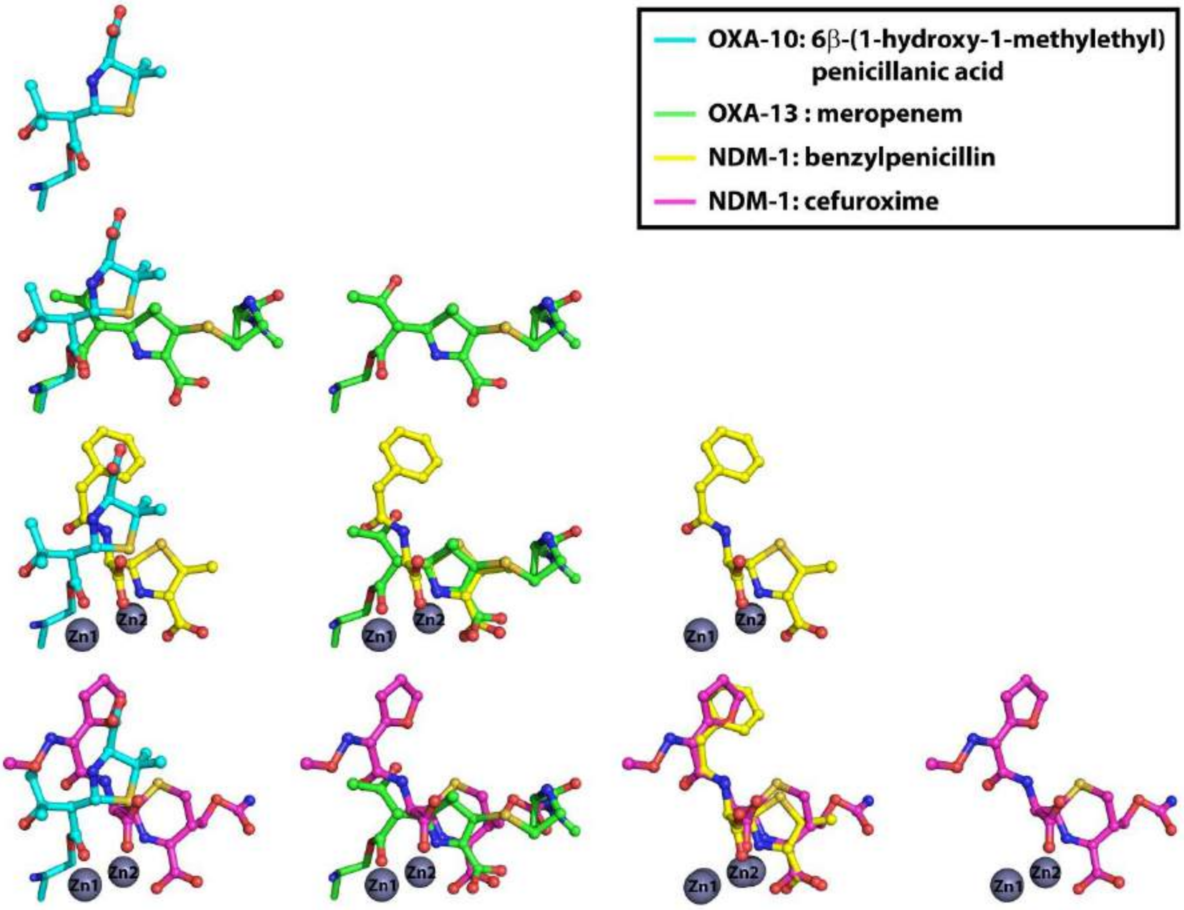

Figure S9: The diversity among conformations of β-lactamase:β-lactam intermediate and product complexes. Overlays of OXA-10:6β-(1-hydroxy-1-methylethyl penicillanic acid(blue), OXA-13:meropenem (green), NDM-1:benzylpenicillin (yellow), and NDM-1:cefuroxime complexes (pink) (PDB accession codes 1K54, 1H8Y, 4EYF and 4RL0, respectively).

Supplemental Disc Diffusion Test Images: Plate Plan

CAR: Carbenacillin PRL: Piperacillin TZP: Piperacillin/Tazobactam KF: Cephalothin

C/T: Ceftolazone/Tazobactam

TEM: Temocillin MEL: Mecillinam CTX: Cefotaxime CAZ: Ceftazidime

SAM:Ampicillin/Sulbactam

CPT: Ceftaroline FEP: Cefipime AZT: Aztreonam IPM: Imipenem

CAZ/AVI: Ceftazidime/Avibactam

AUG: Amoxicillin/Clavulanate ERT: Ertapenem FOX: Cefoxitin MEM: Meropenem

Escherichia coli EC107: CTX-M-15 and OXA-1

β-lactam (μg)

Activity enhanced by **2**

+ **2** 2:1 ratio

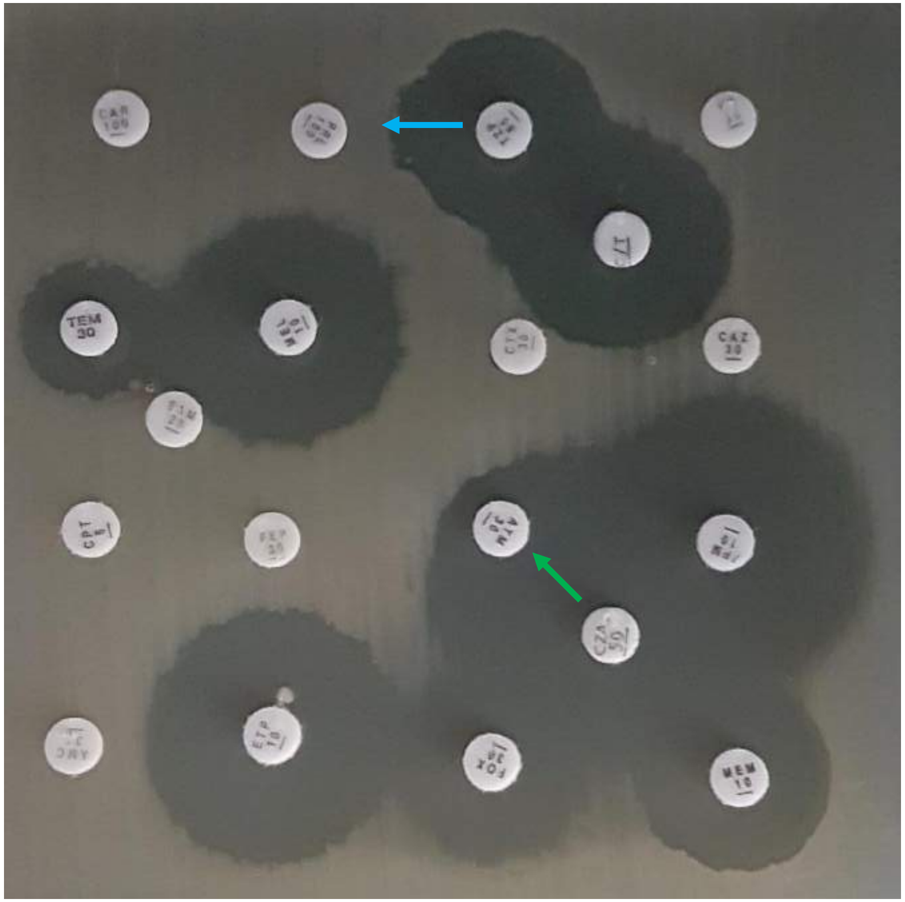

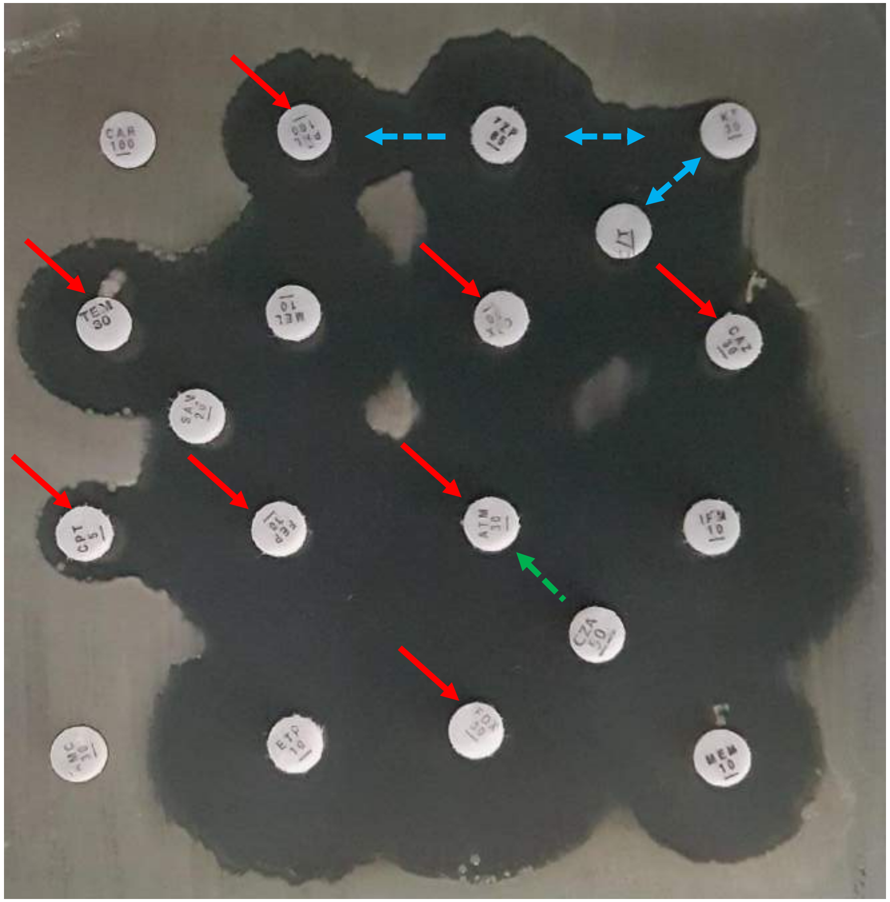

Synergy with Avibactam

Synergy with Clavulanate

Synergy with Tazobactam

Synergy with Sulbactam

Synergy with Avibactam / **2**

Synergy with Clavulanate / **2**

Synergy with Tazobactam / **2**

Synergy with Sulbactam / **2**

Escherichia coli EC114: TEM-1, CTX-M-15 and OXA-1

	β-lactam (μg)	Activity enhanced by **2**
		+ **2** 2:1 ratio

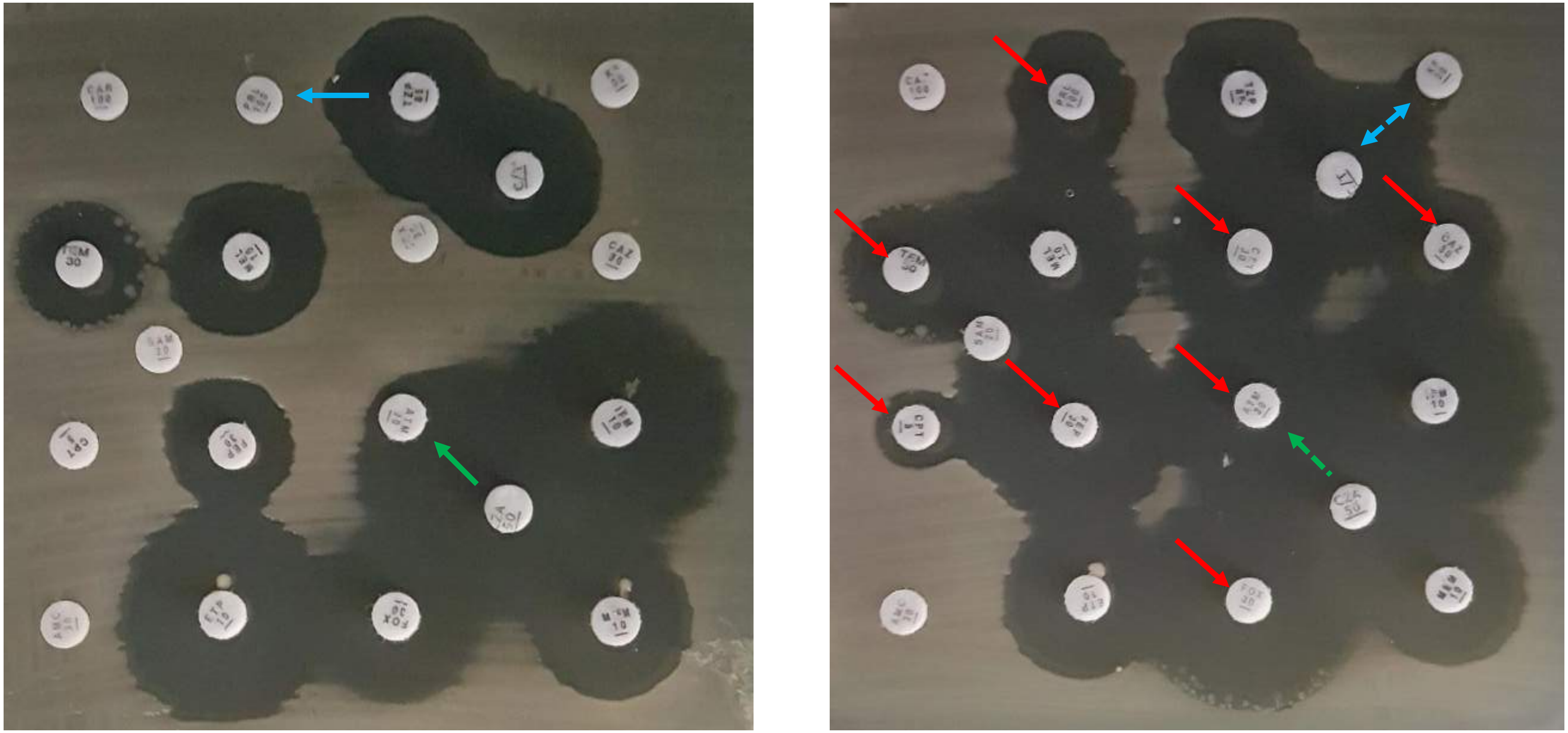

Synergy with Avibactam

Synergy with Clavulanate

Synergy with Tazobactam

Synergy with Sulbactam

Synergy with Avibactam / **2**

Synergy with Clavulanate / **2**

Synergy with Tazobactam / **2**

Synergy with Sulbactam / **2**

Escherichia coli EC86: CTX-M-15,CMY-4 and OXA-181

β-lactam (µg)

Activity enhanced by **2**

+ **2** 2:1 ratio

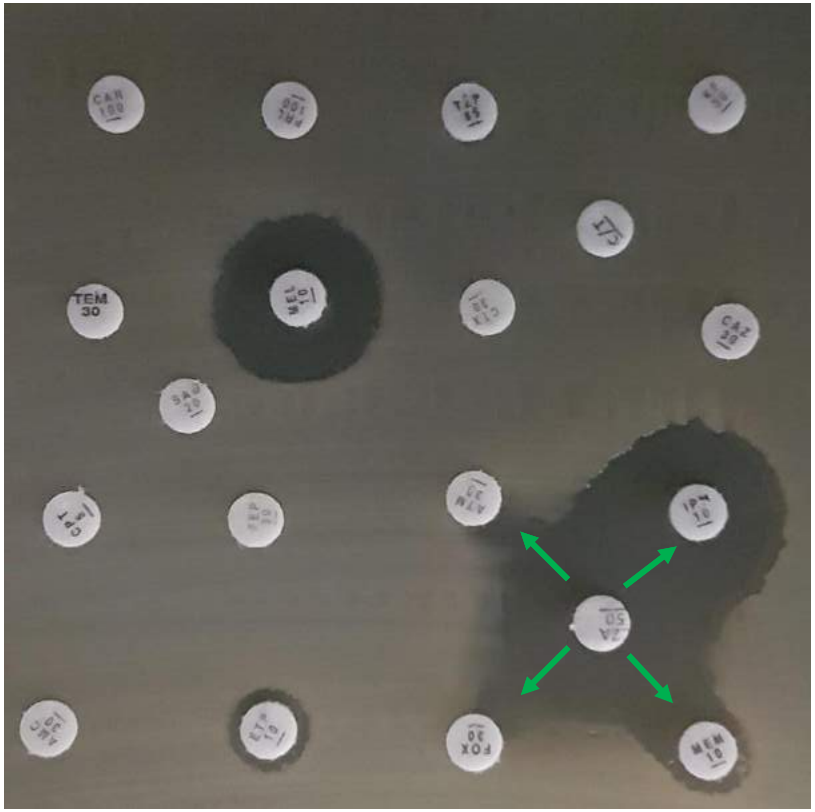

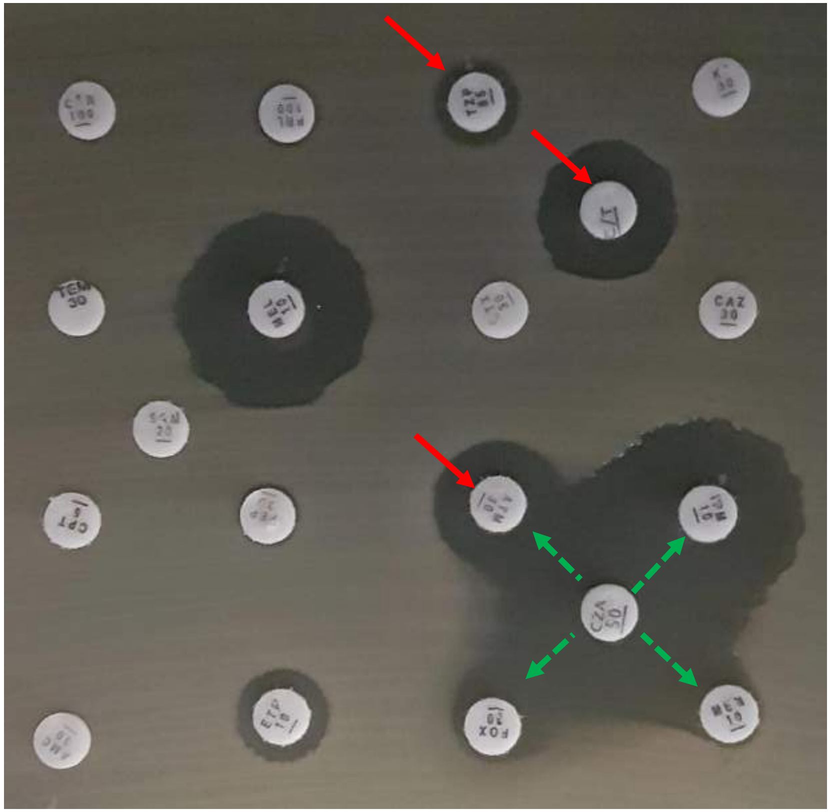

Synergy with Avibactam

Synergy with Clavulanate

Synergy with Tazobactam

Synergy with Sulbactam

Synergy with Avibactam / **2**

Synergy with Clavulanate / **2**

Synergy with Tazobactam / **2**

Synergy with Sulbactam / **2**

Escherichia coli EC113: CTX-M-27

β-lactam (μg)

Activity enhanced by **2**

+ **2** 2:1 ratio

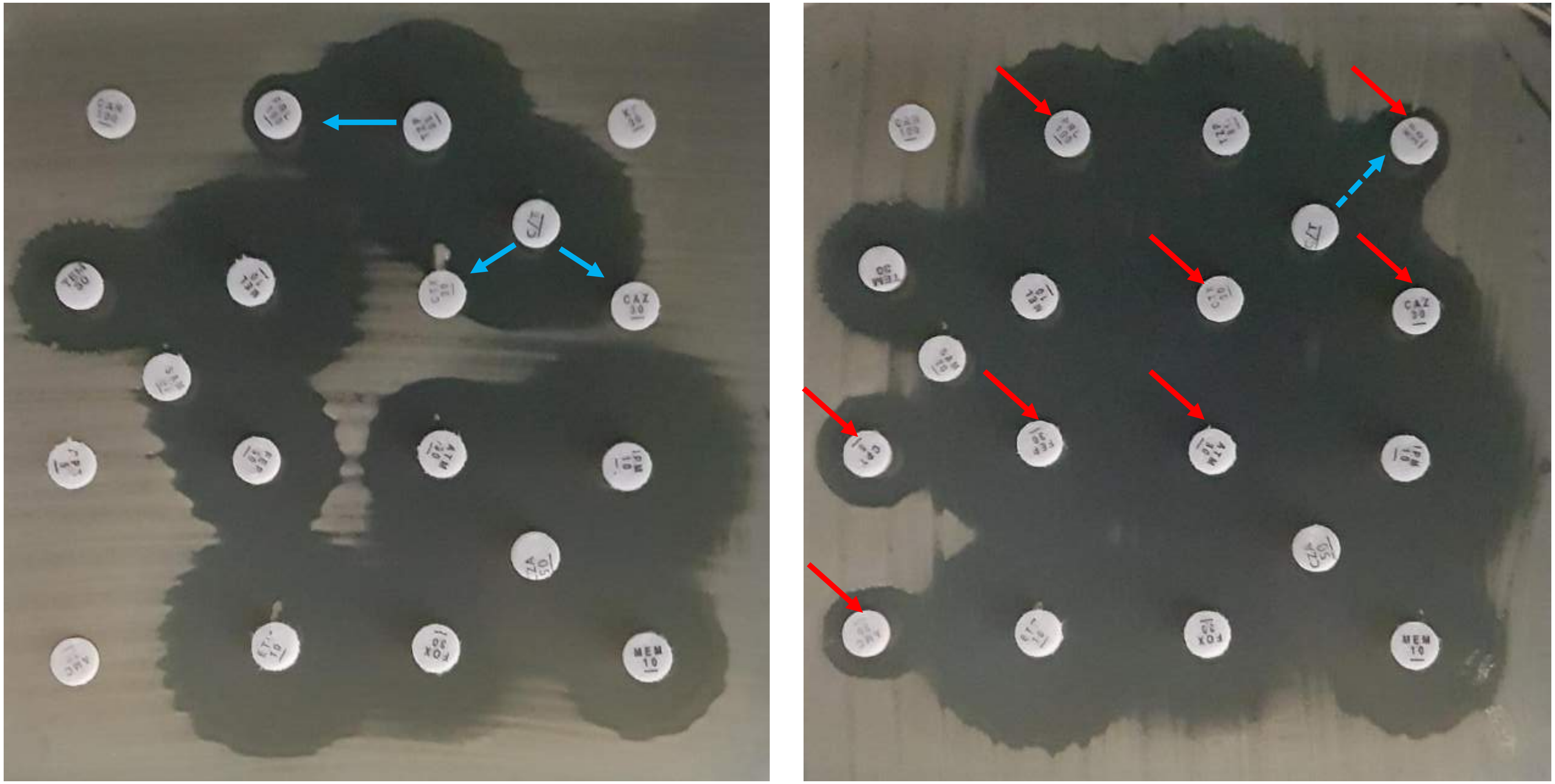

Synergy with Avibactam

Synergy with Clavulanate

Synergy with Tazobactam

Synergy with Sulbactam

Synergy with Avibactam / **2**

Synergy with Clavulanate / **2**

Synergy with Tazobactam / **2**

Synergy with Sulbactam / **2**

Klebsiella pneumoniae KP15: TEM-1, SHV-11, KPC-2

β-lactam (μg)

Activity enhanced by **2**

+ **2** 2:1 ratio

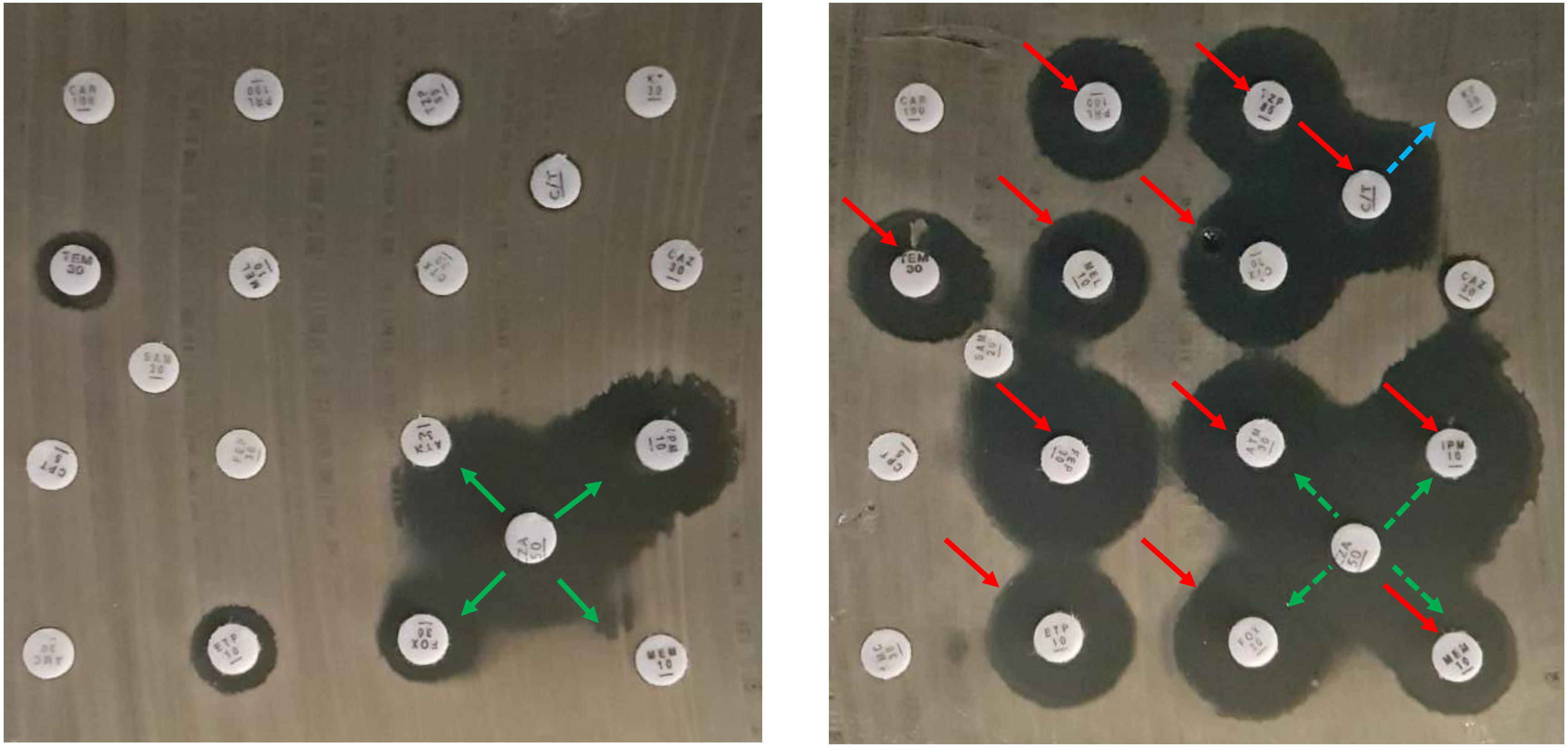

Synergy with Avibactam

Synergy with Clavulanate

Synergy with Tazobactam

Synergy with Sulbactam

Synergy with Avibactam / **2**

Synergy with Clavulanate / **2**

Synergy with Tazobactam / **2**

Synergy with Sulbactam / **2**

Klebsiella pneumoniae KP41:TEM-1, SHV-1,5,11, CTX-M-15, OXA-232

β-lactam (μg)

Activity enhanced by **2**

\+ **2** 2:1 ratio

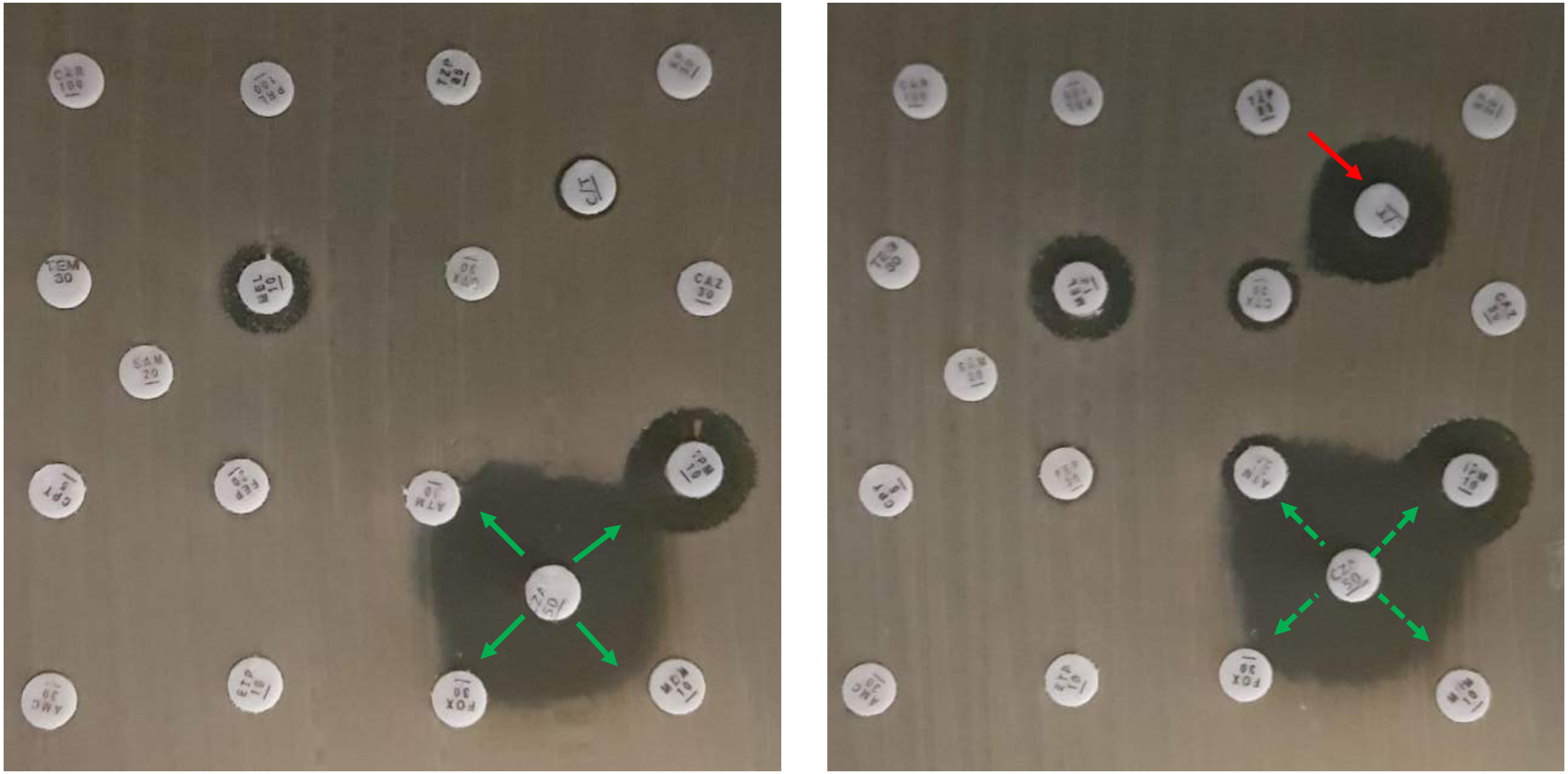

Synergy with Avibactam

Synergy with Clavulanate

Synergy with Tazobactam

Synergy with Sulbactam

Synergy with Avibactam / **2**

Synergy with Clavulanate / **2**

Synergy with Tazobactam / **2**

Synergy with Sulbactam / **2**

Klebsiella pneumoniae KP58: SHV-11, VIM-4

β-lactam (μg) Activity enhanced by **2**

+ **2** 2:1 ratio

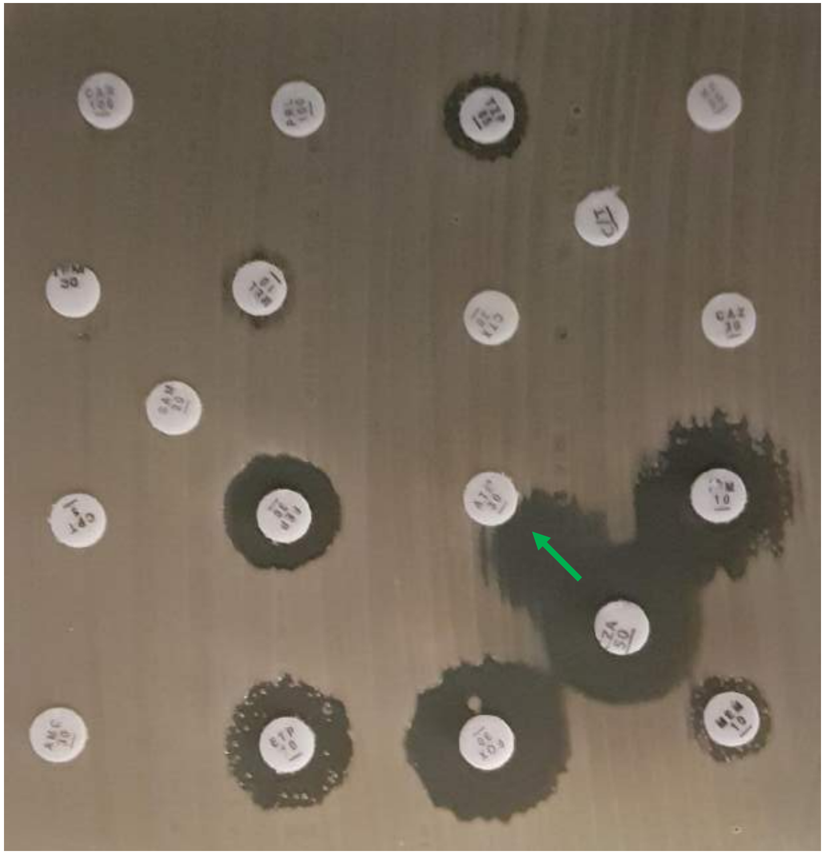

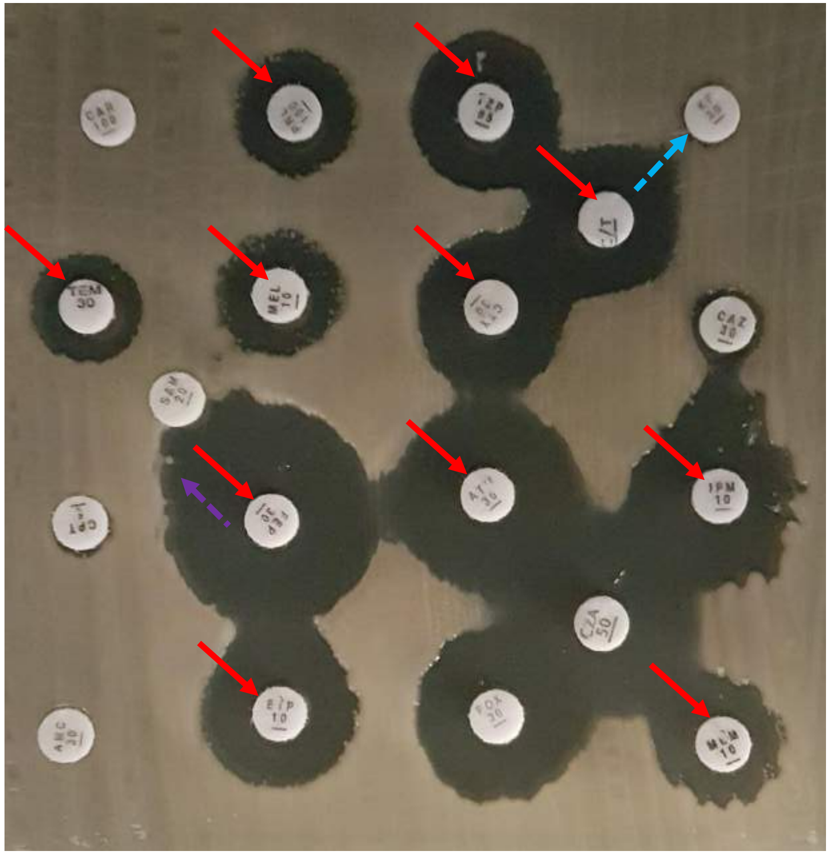

Synergy with Avibactam

Synergy with Clavulanate

Synergy with Tazobactam

Synergy with Sulbactam

Synergy with Avibactam / **2**

Synergy with Clavulanate / **2**

Synergy with Tazobactam / **2**

Synergy with Sulbactam / **2**

Providencia stuartii PS71: TEM-1, SHV-5, VEB-1, VIM-1

β-lactam (μg) | Activity enhanced by **2**

\+ **2** 2:1 ratio

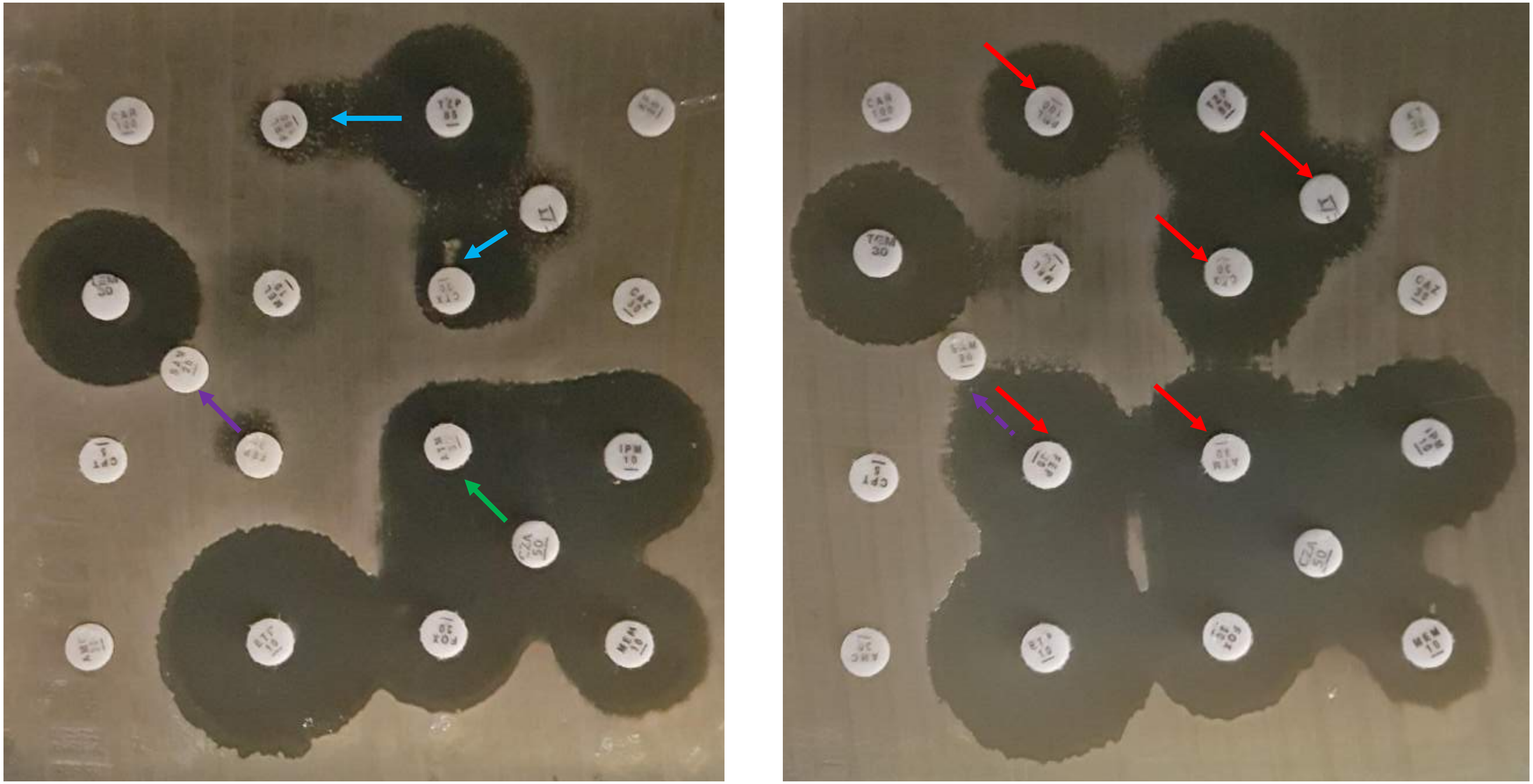

Synergy with Avibactam

Synergy with Clavulanate

Synergy with Tazobactam

Synergy with Sulbactam

Synergy with Avibactam / **2**

Synergy with Clavulanate / **2**

Synergy with Tazobactam / **2**

Synergy with Sulbactam / **2**

Pseudomonas aeruginosa PA12: VIM-2

β-lactam (μg) | Activity enhanced by **2**

+ **2** 2:1 ratio

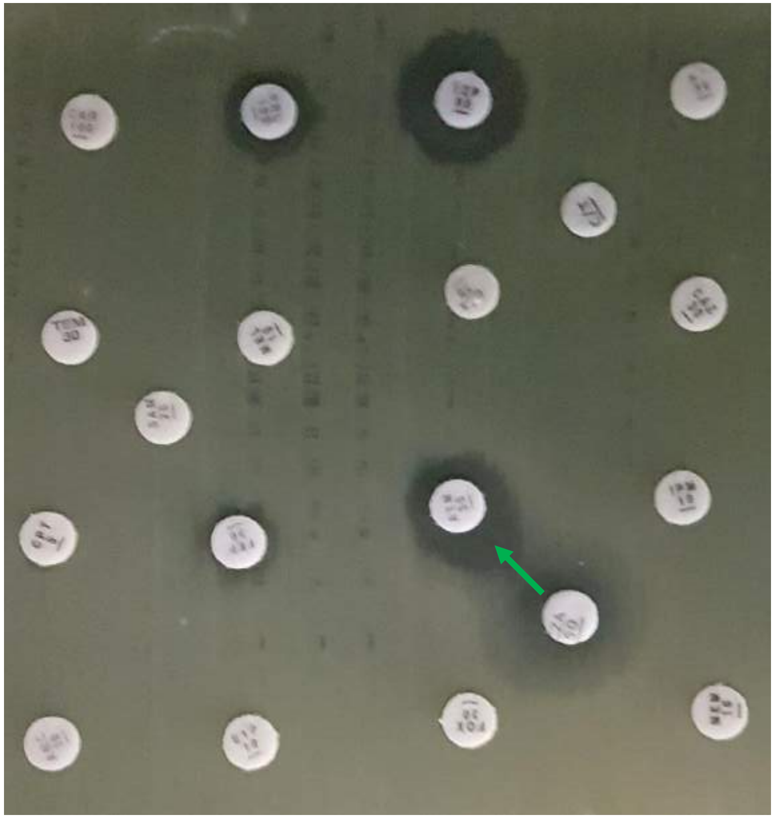

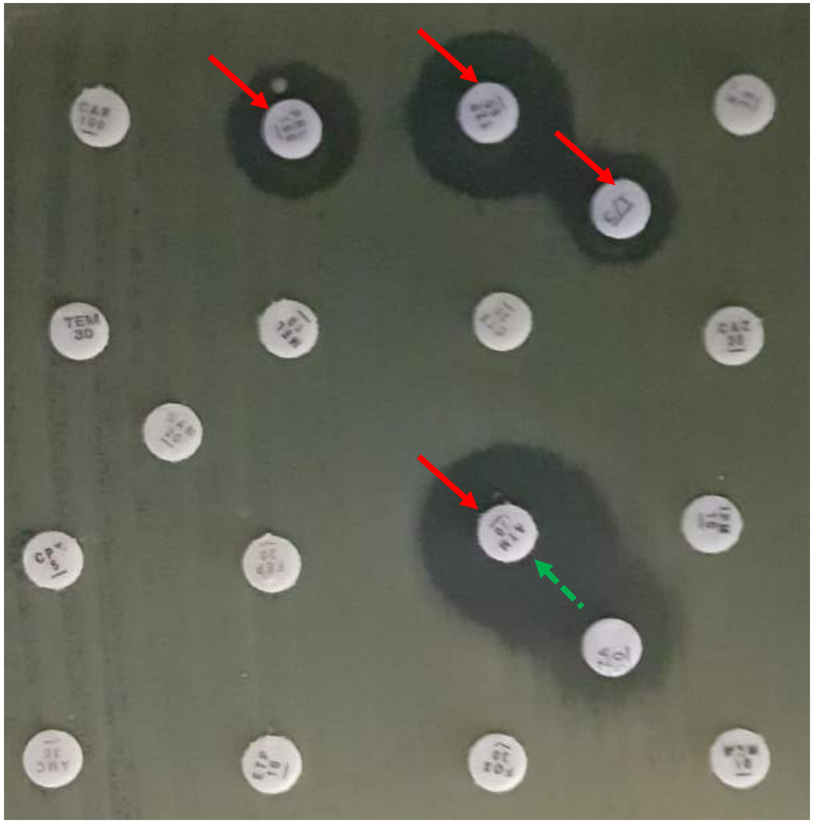

Synergy with Avibactam

Synergy with Clavulanate

Synergy with Tazobactam

Synergy with Sulbactam

Synergy with Avibactam / **2**

Synergy with Clavulanate / **2**

Synergy with Tazobactam / **2**

Synergy with Sulbactam / **2**

Acinetobacter baumannii AB14: OXA-51, OXA-23

β-lactam (μg) | Activity enhanced by **2**

+ **2** 2:1 ratio

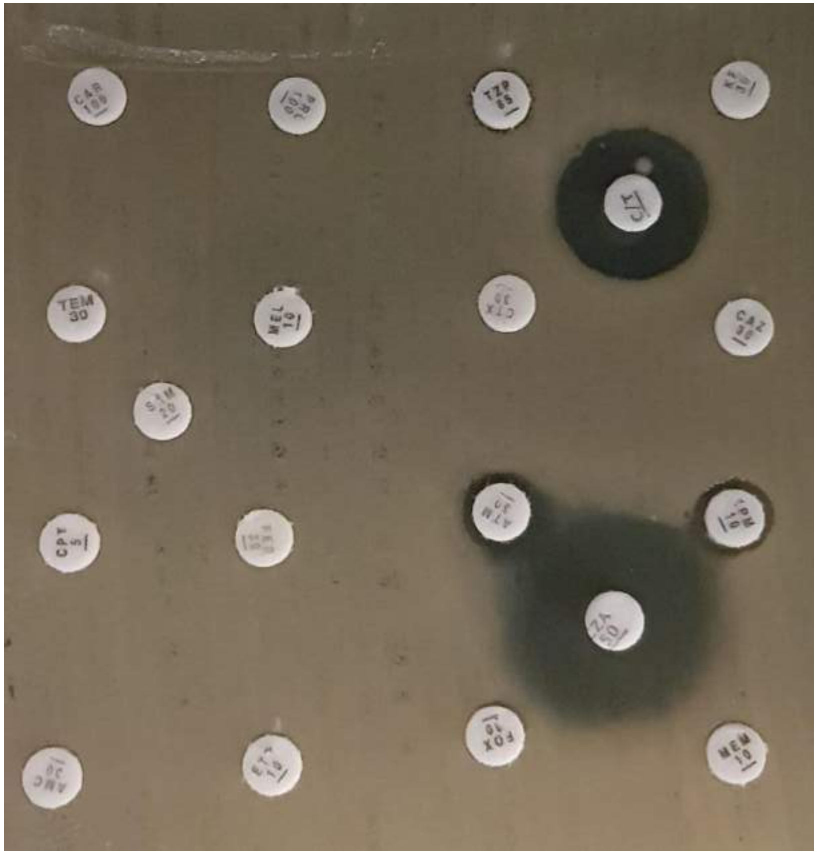

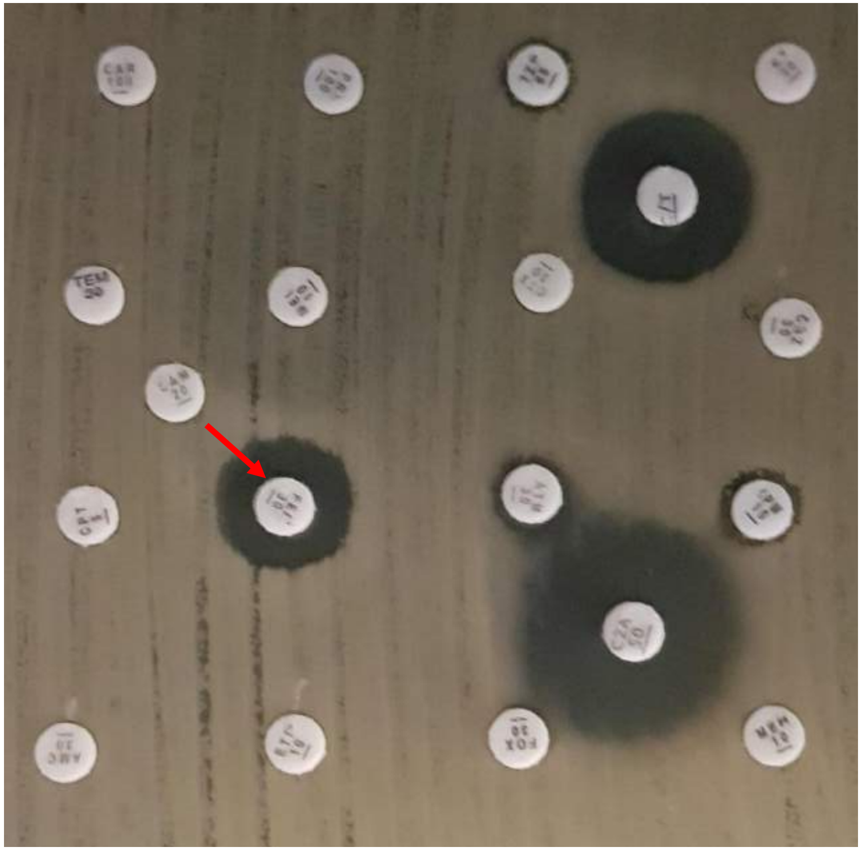

Synergy with Avibactam

Synergy with Clavulanate

Synergy with Tazobactam

Synergy with Sulbactam

Synergy with Avibactam / **2**

Synergy with Clavulanate / **2**

Synergy with Tazobactam / **2**

Synergy with Sulbactam / **2**

References

1. **van Berkel SS, Brem J, Rydzik AM, Salimraj R, Cain R, Verma A, Owens RJ, Fishwick CWG, Spencer J, Schofield CJ.** 2013. Assay Platform for Clinically Relevant Metallo-β-Lactamases. J Med Chem **56:**6945-6953.
2. **GraphPadPrism.** v5.04. GraphPad Software, San Diego, California, USA. www.graphpad.com.
3. **O'Callaghan CH, Morris A, Kirby SM, Shingler AH.** 1972. Novel Method for Detection of β-Lactamases by Using a Chromogenic Cephalosporin Substrate. Antimicrob Agents Chemother **1:**283-288.
4. **Hecker SJ, Reddy KR, Totrov M, Hirst GC, Lomovskaya O, Griffith DC, King P, Tsivkovski R, Sun D, Sabet M, Tarazi Z, Clifton MC, Atkins K, Raymond A, Potts KT, Abendroth J, Boyer SH, Loutit JS, Morgan EE, Durso S, Dudley MN.** 2015. Discovery of a Cyclic Boronic Acid β-Lactamase Inhibitor (RPX7009) with Utility vs Class A Serine Carbapenemases. J Med Chem **58:**3682-3692.
5. **Ness S, Martin R, Kindler AM, Paetzel M, Gold M, Jensen SE, Jones JB, Strynadka NCJ.** 2000. Structure-Based Design Guides the Improved Efficacy of Deacylation Transition State Analogue Inhibitors of TEM-1 β-Lactamase. Biochemistry **39:**5312-5321.
